# Holistic determination of ends of cfDNA molecules

**DOI:** 10.1016/j.xgen.2026.101142

**Published:** 2026-02-06

**Authors:** Peiyong Jiang, Mary-Jane L. Ma, Rong Qiao, Yuwei Shi, Jing Liu, Qing Zhou, Wenlei Peng, W.K. Jacky Lam, Jinyue Bai, L.Y. Lois Choy, W.H. Adrian Tsui, Yasine Malki, Guannan Kang, Stephanie C.Y. Yu, Dongyan Xiong, Grace L.H. Wong, Landon L. Chan, John Wong, Stephen L. Chan, Vincent W.S. Wong, K.C. Allen Chan, Y.M. Dennis Lo

**Affiliations:** 1 Centre for Novostics, Hong Kong Science Park, Pak Shek Kok, New Territories, Hong Kong SAR, China; 2 Li Ka Shing Institute of Health Sciences, the Chinese University of Hong Kong, Shatin, New Territories, Hong Kong SAR, China; 3 Department of Chemical Pathology, the Chinese University of Hong Kong, Prince of Wales Hospital, Shatin, New Territories, Hong Kong SAR, China; 4 State Key Laboratory of Translational Oncology, the Chinese University of Hong Kong, Prince of Wales Hospital, Shatin, Hong Kong SAR, China; 5 Department of Medicine and Therapeutics, the Chinese University of Hong Kong, Prince of Wales Hospital, Shatin, Hong Kong SAR, China; 6 Department of Clinical Oncology, Sir Y. K. Pao Centre for Cancer, the Chinese University of Hong Kong, Prince of Wales Hospital, Shatin, Hong Kong SAR, China; 7 Department of Surgery, the Chinese University of Hong Kong, Prince of Wales Hospital, Shatin, Hong Kong SAR, China

**Keywords:** fragmentomics, end motifs, noninvasive, cancer detection

## Abstract

Cell-free DNA (cfDNA) end motifs serve as fragmentomics biomarkers for cancer. Prior studies primarily focused on 5′ ends, whereas 3′ ends were overlooked due to artifactual modification in existing sequencing protocols. We utilized single-stranded library preparation (“2-end sequencing”) to assess the native 5′ and 3′ end motifs (EM5 and EM3, respectively). Additionally, we demonstrated diagnostic power from the nucleotide motifs located immediately upstream and downstream of 5′ and 3′ ends, named pre-end motifs (PREMs) and post-end motifs (POEMs). These fragmentomics markers collectively achieved an area under the curve (AUC) of 0.95 for hepatocellular carcinoma (HCC) detection. Fragmentomics-based methylation analysis of 3′ ends (3′ FRAGMA) improved detection of HCC (AUC: 0.97). We further developed “4-end sequencing” to interrogate both ends of both strands of a double-stranded cfDNA molecule, enhancing fragmentomics-based cancer detection. Holistic end profiling adds to the armamentarium of liquid biopsy and sheds light on the biology of cfDNA fragmentation.

## Highlights

• ssDNA sequencing library preparation enables 3′ fragmentomics analysis of cfDNA
• Stem-loop-adaptor-mediated dsDNA sequencing enables holistic analysis of all four ends
• A constellation of 5′, 3′, pre-, and post-end fragmentomics markers is developed
• Holistic ends analysis boosts cancer detection and deepens cfDNA biological insights

## Graphical abstract

**Figure:**
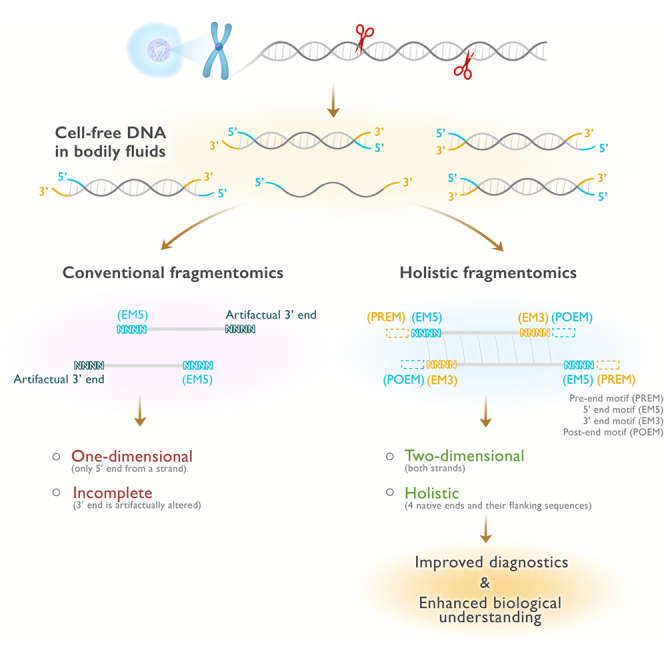

## Teaser

cfDNA fragmentomics is an emerging branch of liquid biopsy. Jiang et al. broke through the conventional limitation of 5′ fragmentomics to a constellation of markers involving the 3′ end, sequences flanking fragmentation sites, and all four ends of a dsDNA molecule. Holistic fragmentomics enhances diagnostic performance and deepens biological insights.

## Introduction

Fragmentomics of cell-free DNA (cfDNA) in bodily fluids such as plasma is a rapidly advancing field of research.^1^^,^^2^^,^^3^^,^^4^ Many studies focused on fragmentation patterns of cfDNA molecules, such as fragment sizes,^5^^,^^6^^,^^7^^,^^8^^,^^9^^,^^10^ preferred ends,^11^^,^^12^ end motifs,^13^^,^^14^^,^^15^^,^^16^^,^^17^ nucleosomal patterns,^18^^,^^19^ jagged ends,^20^^,^^21^ and long^22^^,^^23^ and ultra-short^24^^,^^25^^,^^26^^,^^27^^,^^28^ cfDNA populations. For instance, it has been revealed that a population of genomic coordinates exhibited significant overrepresentation at the 5′ ends of cfDNA fragments, referred to as preferred end coordinates. cfDNA molecules that terminate at preferred sites possess valuable tissue-of-origin information associated with their ends.^11^^,^^12^ Subsequent studies have demonstrated that the analysis of short stretches of nucleotides at cfDNA fragment ends (e.g., 5′ 4-mer end motifs) holds significant potential for the detection of patients with cancers such as hepatocellular carcinoma (HCC), renal cell carcinoma, colon adenocarcinoma, etc.^13^^,^^15^ The efficacy of cancer detection using these end motifs may be linked to cancer-associated alterations in enzymatic processes during apoptosis.^13^ The “CCCA” end motif was significantly decreased in deoxyribonuclease 1-like 3 (*Dnase1l3*) knockout mice compared to their wild-type counterparts.^17^ This observation aligns with findings in patients suffering from various cancers, where a reduction in “CCCA” end motifs was associated with the downregulation of *DNASE1L3* across those cancer types.^13^ DNA nucleases are also implicated in cfDNA fragmentation across a wide range of conditions, including Epstein-Barr virus (EBV) persistence in individuals, pregnancy, and bacterial infections in septic patients.^29^^,^^30^^,^^31^

However, previous studies on cfDNA fragmentomics have mainly focused on elucidating the 5′ ends of cfDNA fragments. This focus is largely attributable to the widespread use of sequencing library preparation that is designed for analyzing double-stranded DNA (dsDNA) molecules. Such dsDNA library preparation involves an end-repair process that removes the 3′ protruding single-stranded ends and elongates the 3′ recessed ends using the opposite 5′ protruding single strand as a DNA template. As a result, the intrinsic characteristics of the 3′ ends are lost or altered, and their potential diagnostic value has remained unexplored.

Recently, single-stranded DNA (ssDNA) library preparation^32^ has been employed to study cfDNA molecules.^24^^,^^33^ Unlike dsDNA library preparation, ssDNA library preparation ligates the sequencing adapter directly to single-stranded molecules after DNA denaturation, preserving both 5′ and 3′ native ends. We reasoned that this approach would theoretically provide an opportunity to explore 3′ end information. However, previous studies based on ssDNA library preparation have not investigated the diagnostic relevance of native 3′ ends.

We hypothesized that the 3′ ends might contain valuable diagnostic information and that integrating data from both the 5′ and 3′ ends could enhance the clinical and biological utility of cfDNA fragmentomics. In this work, we thus proposed a holistic determination of cfDNA ends and evaluated its potential for clinical applications. We first employed ssDNA library preparation to investigate the directly measured native 5′ and 3′ end motifs derived from individual strands (referred to as EM5 and EM3, respectively), together with their deduced upstream and downstream end motifs of a sequenced fragment aligned to the reference genome. This strategy for analyzing EM5 and EM3 is referred to as “2-end sequencing.” The deduced end motifs upstream of the 5′ end are referred to as PREMs (pre-end motifs), while the deduced end motifs downstream of the 3′ end are referred to as POEMs (post-end motifs). However, since two strands of a dsDNA molecule are sequenced separately and independently on the Illumina platform, the combinatory information of the four native ends from both strands of the same molecule cannot be directly resolved. Therefore, we further developed a sequencing protocol that enabled the simultaneous assessment of all four termini of a native double-stranded cfDNA molecule (referred to as “4-end sequencing”).

**Figure 1:**
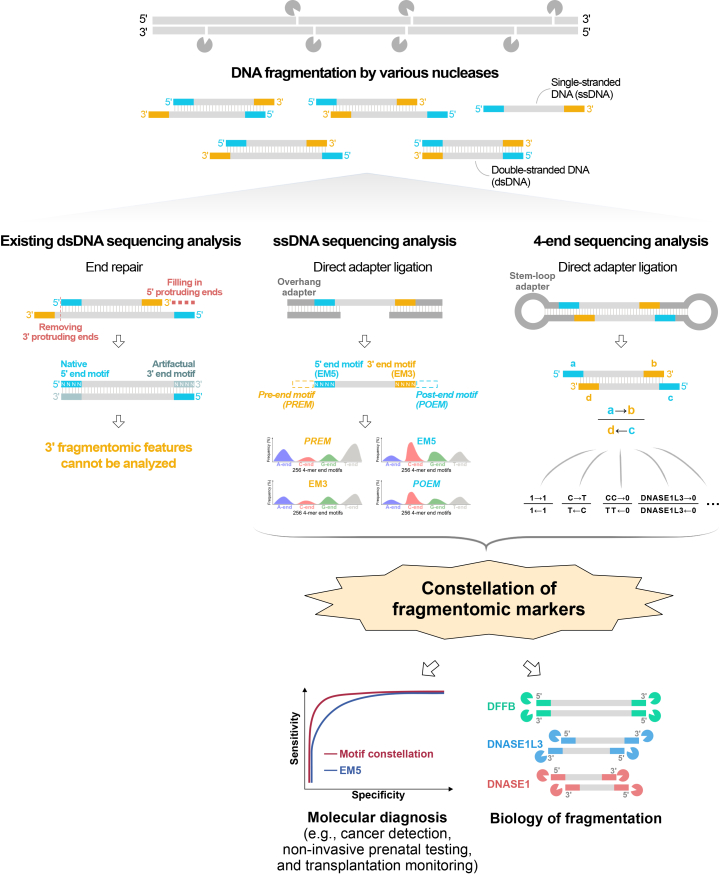
Schematic of the holistic analysis of end motifs of cfDNA molecules Circulating DNA in plasma consists of a mixture of single-stranded (ss) and double-stranded (ds) DNA fragments. The existing dsDNA library preparation involves an end-repair process that removes the 3′ protruding single-stranded ends and elongates the 3′ recessed ends using the opposite 5′ protruding single strand as a DNA template. As a result, the intrinsic characteristics of the 3′ ends are lost or altered, and their potential diagnostic value has remained unexplored. In this study, we developed two approaches for assessing the molecular properties of native ends of cfDNA fragments, namely, 2-end sequencing and 4-end sequencing. For 2-end sequencing, we adapted ssDNA library preparation for the Illumina sequencing platform, which involved DNA denaturation followed by direct adapter ligation but omitting the DNA end-repair process. This method thus preserves the original end information of both ssDNA and dsDNA fragments in sequencing data. From 2-end sequencing results, the 5′ and 3′ end motifs that are directly measured from individual strands are referred to as EM5 and EM3. Since the footprint of DNA nucleases acting on the cfDNA fragmentation may involve several nucleotides surrounding the cleavage sites, we also analyzed the end motifs located upstream of the 5′ end (PREM [pre-end motif]) and downstream of the 3′ end (POEM [post-end motif]), which were inferred from the reference genome. The DNA denaturation step in ssDNA library preparation separates dsDNA into individual strands, thereby preventing the simultaneous capture of end information from both strands. To address this limitation, we developed an experimental protocol (4-end sequencing), enabling the simultaneous assessment of all four termini of a native dsDNA molecule. In this approach, dsDNA fragments are directly ligated with stem-loop adapters. These stem-loop adapters are engineered with customized end structures featuring random single-stranded overhangs of varying lengths, thus facilitating hybridization to the complementary native ends of cfDNA fragments. Only circularized products with successful ligation at all four termini are eligible for sequencing on the Pacific Biosciences (PacBio) single-molecule real-time (SMRT) sequencing platform. The composition of each overhang corresponds to a specific barcode sequence within the stem-loop adapter, which can be traced in the sequencing data. These previously unrecognized end motifs revealed by these two approaches hold promise for applications in molecular diagnosis (e.g., noninvasive cancer detection) and advancing our understanding of cfDNA biology.

We analyzed these fragmentomics markers within the context of oncology, exploring their potential augmentation of the diagnostic power for cfDNA-based liquid biopsy and for studying the biology of cfDNA fragmentation. The overall study design is schematically illustrated in Figure 1.

## Results

### cfDNA analysis using ssDNA library preparation reveals previously unrecognized fragmentomics markers

To assess fragment sizes and native end motifs for plasma DNA, we adapted ssDNA library preparation followed by paired-end sequencing on the Illumina platform, referred to as 2-end sequencing in this study. Briefly, plasma DNA underwent denaturation, converting dsDNA molecules into single-stranded forms. The resulting single-stranded cfDNA fragments were subjected to direct ligation of sequencing adapters, allowing DNA strands to be sequenced individually (see details in STAR Methods). This 2-end sequencing thus circumvented the conventional end-repair process required in dsDNA library preparation, preserving the native ends of individual single-stranded fragments.

We analyzed 38 healthy controls, with a median of 46 million paired-end reads (range: 26–58 million). The size distribution of plasma DNA derived from ssDNA library preparation displayed two prominent peaks at approximately 52 and 166 nt, with observable but weakly oscillating 10-nt periodicities between these peaks. This finding was in agreement with several previous reports.^24^^,^^25^^,^^26^^,^^27^^,^^28^ In contrast, for dsDNA library preparation, only one predominant peak was at 166 bp, with a series of 10-bp oscillations for molecules below 150 bp (Figure S1A). In this study, when referring to ssDNA, the unit “nt” was used. In contrast, when discussing dsDNA, “bp” was used.

We further investigated the end motifs of cfDNA. We hypothesized that DNA nucleases responsible for cleaving the genome to generate fragmented cfDNA molecules might interact with several nucleotides flanking the cleavage sites. The sequence motifs immediately adjacent to the sequenced ends were likely engaged in the interaction between the nucleases and the DNA being cut. Hence, we interrogated EM5 and EM3, as well as the sequence motifs adjacent to EM5 and EM3, which were deduced from the reference genome. The sequence motif preceding the measured EM5 was termed PREM, and the motif following EM3 was termed POEM (Figure 1). In this analysis, we determined 4-mer end motifs for PREM, EM5, EM3, and POEM in a 5′-to-3′ direction and calculated each of their frequencies.

**Figure 2:**
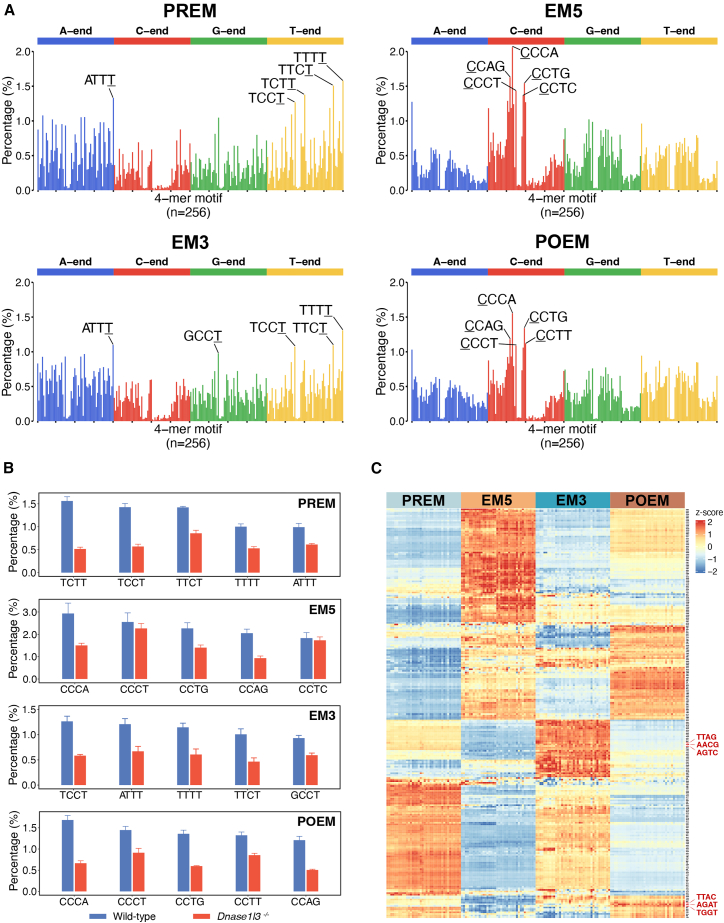
End motif analysis for plasma DNA of healthy controls using 2-end sequencing (A) End-motif profiles of plasma cfDNA molecules from healthy human controls (*n* = 38) in terms of PREM, EM5, EM3, and POEM. Those 256 motifs are arranged in alphabetical order. For each type of motif, the top 5 motifs with the highest motif frequencies are highlighted. (B) Bar charts comparing motif frequencies between *Dnase1l3*-knockout mice (*n* = 5) and matched wild-type mice (*n* = 4) for studying the roles of the DNA nuclease acting on the top 5 motifs identified from healthy human controls (*n* = 38). In this bar plot, each bar represents the mean value, while the whiskers indicate one standard deviation (SD) from the mean. (C) Heatmap analysis of the 256 motif frequencies across PREM, EM5, EM3, and POEM using all plasma DNA fragments in healthy controls (*n* = 38). Each column represents a plasma DNA sample subjected to a certain type of end-motif analysis, and each row represents a 4-mer end motif. For better visualization, row-wise normalization (*Z* score) was applied to motif frequencies. For each motif row, the mean (*m*) and SD (*s*) of motif frequencies were computed across all individuals. Each individual motif frequency (*f*) was then standardized into a *Z* score (*z*) using the formula: *z* = (*f* – *m*)/*s*. Higher frequencies are represented in red, whereas lower frequencies are shown in blue.

Figure 2A shows the frequencies of 256 end motifs in the sequenced results pooled from 38 healthy human control samples for PREM, EM5, EM3, and POEM, respectively. The 256 end motifs are organized in alphabetical order. Motifs starting with adenine (A), cytosine (C), guanine (G), and thymine (T) are highlighted in blue, red, green, and yellow, respectively. We identified the top five motifs for PREM, EM5, EM3, and POEM as (TTTT, TTCT, TCTT, ATTT, TCCT), (CCCA, CCAG, CCTG, CCCT, CCTC), (TTTT, ATTT, TTCT, TCCT, GCCT), and (CCCA, CCTG, CCAG, CCTT, CCCT), respectively. The 4-mer motif is written in a 5′-to-3′ direction, and the base nearest to the cleavage site is underlined. For example, the EM3 motif “ATTT” corresponds to the sequence 5′ ATTT 3′, where the terminal nucleotide at the 3′ end is “T.” Notably, the top EM5 and POEM predominantly began with cytosine (C) at the 5′ end. In contrast, the top EM3 and PREM were characterized by thymine (T) at the 3′ end, suggesting distinct cleavage preferences between motif classes. The top five ranked motifs for PREM, EM5, EM3, and POEM accounted for 7.06%, 8.03%, 5.55%, and 6.29% of the total sequenced molecules, respectively. Notably, those top motifs were generally significantly downregulated in *Dnase1l3*^−/−^ mice compared with wild-type mice. On the other hand, no statistically significant changes were observed for *Dnase1*^−/−^ or *Dffb*^−/−^ mice (Figures 2B, S1B, and S1C). The results suggest that these four classes of end motifs (i.e., PREM, EM5, EM3, and POEM) were primarily generated by DNASE1L3 in plasma.

We used a heatmap to visualize the pattern of PREM, EM5, EM3, and POEM based on their respective 4-mer end motifs (Figure 2C). PREM and EM3 shared noticeable similarities, as indicated by the similar color patterns presented in the heatmap. A minority of discordant patterns were also observed. A similar conclusion could be drawn for EM5 and POEM, with similarities overriding the relatively minor differences. The Pearson’s correlation coefficients (Pearson’s *r*) of PREM versus EM3 and EM5 versus POEM were 0.91 and 0.94, respectively (*p* < 0.001 for both), higher than the Pearson’s *r* values related to other comparisons ranging from 0.28 to 0.53 (PREM versus EM5, 0.28; PREM versus POEM, 0.46; EM5 versus EM3, 0.36; and EM3 versus POEM, 0.53). We found that the top-ranked 20 motifs considerably overlapped between PREM and EM3, as well as between EM5 and POEM, but not in other combinations. Among these top-ranked motifs, 14 out of 20 were shared between EM5 and POEM, and 18 out of 20 were shared between PREM and EM3 (Table S1). In addition to largely similar patterns between PREM and EM3 and between EM5 and POEM, it is worth highlighting the areas of discordance. For example, the 4-mer end motifs TTAG, AACG, and AGTC were differentially represented between PREM and EM3, while TTAC, AGAT, and TGGT significantly differed between EM5 and POEM (Figure 2C).

We further studied how the end-motif patterns were associated with the two peaks approximately occurring at 52 and 166 nt, respectively (Figure S1A). We selected two windows of 32–72 and 146–186 nt, spanning 20 nt upstream and downstream of each peak. The motif patterns for molecules associated with these two size ranges were analyzed. When we applied the heatmap to analyze the plasma DNA populations associated with the 1^st^ peak in the size profile, the patterns among these four types of end motifs became less distinct (Figure S1D). The correlations between PREM and EM3 and between EM5 and POEM decreased to 0.73 and 0.81, respectively. However, for the plasma DNA populations associated with the 2^nd^ peak, the patterns related to these four types of end motifs became distinct again. The Pearson’s *r* between EM5 and POEM and between PREM and EM3 are 0.93 and 0.92, respectively (Figure S1E). Additionally, the Pearson’s *r* values between EM5 and POEM and between PREM and EM3 were lower in the relatively short cfDNA population with a size of 42–70 nt (Pearson’s *r*, 0.79 and 0.71) (Figure S2A), compared with the long cfDNA populations with a size of 70–166 nt (Pearson’s *r*, 0.94 and 0.92) (Figure S2B) and 166–600 nt (Pearson’s *r*, 0.93 and 0.94) (Figure S2C). These observations suggest that the long cfDNA population exhibits stronger correlations between EM5 and POEM, as well as between PREM and EM3, consistent with the patterns observed in size ranges associated with the 1^st^ and 2^nd^ peaks.

Using deconvolutional analysis of EM5 end motifs,^34^ we found that the DNASE1L3 signature (e.g., F-profile I) was significantly reduced in individuals with higher cfDNA concentrations (median, 38.9; range, 36.8–44.7) compared to those with lower concentrations (median, 41.3; range, 35.9–44.8; *p* = 0.022, one-sided Mann–Whitney *U* test) (Figure S3A). We also observed a trend toward a higher proportion of long cfDNA fragments (>166 bp) in the high-concentration group (Figure S3B), although this did not reach statistical significance. These findings generally support the relationship between DNASE1L3 signals and cfDNA concentrations reported by Malki et al.^31^

### Differential end motifs of plasma cfDNA between patients with and without HCC

To explore the diagnostic value of the end motifs identified in this study, we sequenced samples from 38 healthy control subjects (CTRs), 35 patients chronically infected with hepatitis B virus (HBV) but without HCC, and 43 patients with HCC, using ssDNA library preparation. We obtained a median of 46 million paired-end reads (range, 21–58 million).

**Figure 3:**
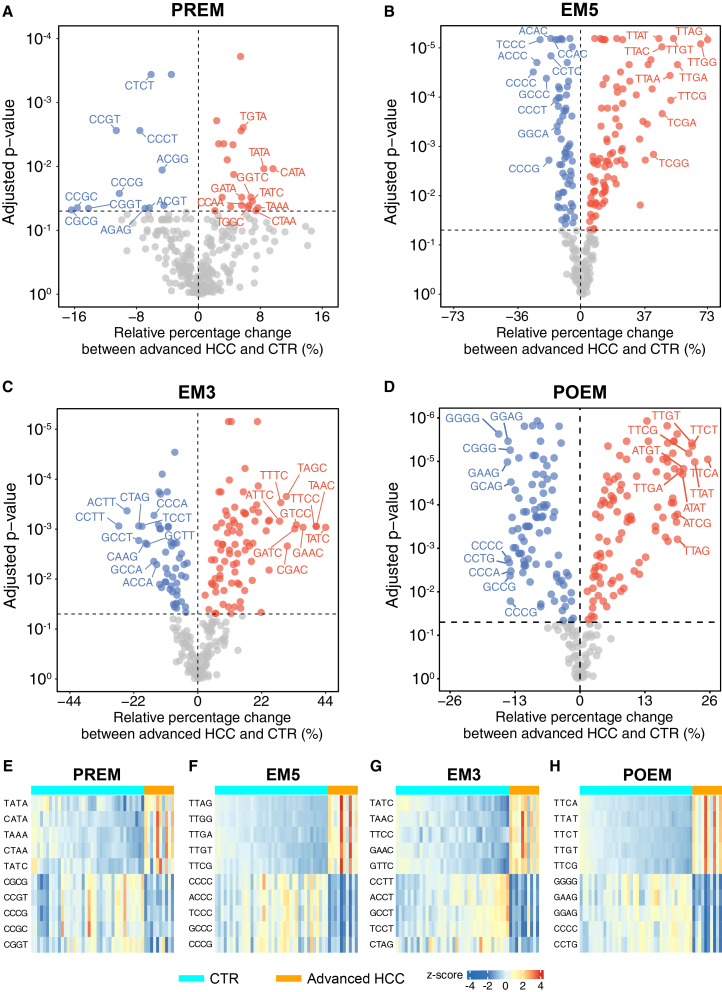
Differential end motifs between advanced HCC and control groups using all plasma DNA fragments (A–D) Volcano plots of differentially increased (red dots) and decreased (blue dots) end motifs in those patients with advanced HCC (*n* = 10) were analyzed for PREM (A), EM5 (B), EM3 (C), and POEM (D), compared with healthy controls (*n* = 38). (E–H) Heatmaps of motif frequencies of the top 5 differentially increased and decreased end motifs (ranked by relative percentage change) between HCC (*n* = 43) and control (*n* = 38) groups were shown for PREM (E), EM5 (F), EM3 (G), and POEM (H).

We identified the differential end motifs between advanced HCC (i.e., Barcelona Clinic Liver Cancer [BCLC] stage C) and healthy control groups (see details in STAR Methods). As shown in Figures 3A–3D, we obtained 34, 158, 139, and 194 differential 4-mer end motifs associated with PREM, EM5, EM3, and POEM, respectively, when using all sequenced fragments (i.e., without *in silico* size selection). The top 5 differential motifs showed distinct patterns in motif frequencies between patients with advanced HCC and CTRs (Figures 3E–3H). For PREM, TATA, CATA, TAAA, CTAA, and TATC end motifs exhibited increased representation in the advanced HCC group, whereas CGCG, CCGT, CCCG, CCGC, and CGGT exhibited decreased representation (Table S2). We further determined an end-motif ratio (EMR) metric, which was derived by calculating the ratio of the cumulative frequency of end motifs that exhibited increased representation in the HCC group to those that exhibited decreased representation. The EMR values were determined for all samples, including those of healthy controls, carriers of HBV, and patients with HCC with different tumor stages. The HCC group had a significantly higher EMR for PREM (median, 2.80; range, 2.54–3.56), in comparison with the non-HCC group (median, 2.70; range, 2.38–2.96) (*p* < 0.0001; two-sided Mann-Whitney *U* test) (Figure S4A). Similarly, we identified the differential end motifs in EM5 (e.g., increased motifs: TTAG, TTGG, TTGA, TTGT, and TTCG; decreased motifs: CCCC, ACCC, TCCC, GCCC, and CCCG), EM3 (e.g., increased motifs: TATC, TAAC, TTCC, GAAC, and GTTC; decreased motifs: CCTT, ACCT, GCCT, TCCT, and CTAG), and POEM (e.g., increased motifs: TTCA, TTAT, TTCT, TTGT, and TTCG; decreased motifs: GGGG, GAAG, GGAG, CCCC, and CCTG). The 4-mer motif is written in a 5′-to-3′ direction, and the base nearest to the cleavage site is underlined. For these types of end motifs, we also observed that EMR values were significantly elevated in the HCC group compared to the non-HCC group (Figures S4B–S4D). These results indicate that these identified end motifs have potential for cancer detection.

### Combined analysis of various end motifs for differentiating patients with and without HCC

**Figure 4:**
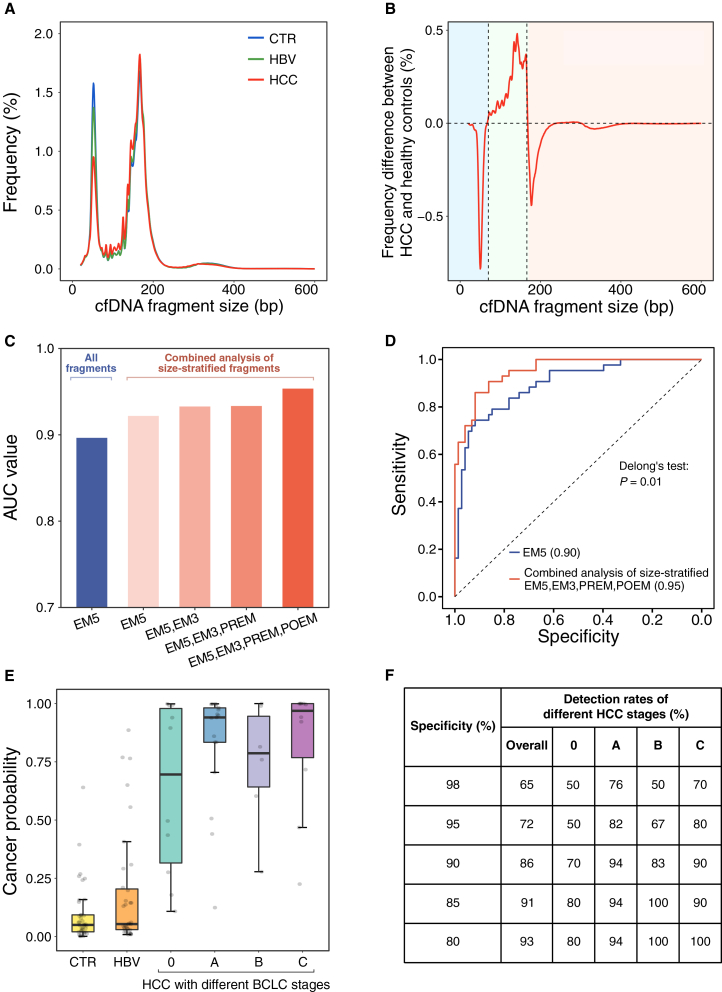
Combined analysis of size-stratified end motifs for HCC detection (A) Size profiles of pooled sequencing results from healthy control subjects (CTRs) (*n* = 38), carriers of HBV (*n* = 35), and patients with HCC (*n* = 43), respectively. (B) Differences in size frequencies between a representative patient with HCC with the highest tumor DNA fraction and the median size profile of the healthy control group. (C) Barplot of AUC values for various analytical strategies utilizing PREM, EM5, EM3, and POEM features. (D) Performance comparison of the traditional EM5 analysis and the combined analysis of size-stratified PREM, EM5, EM3, and POEM features. *P* value was calculated using Delong's test. (E) Boxplot of probabilities of having cancer using the combined analysis of size-stratified PREM, EM5, EM3, and POEM features. In this boxplot, the central line shows the median value. The bottom and top edges of the box represent the 25^th^ (Q1) and 75^th^ (Q2) percentiles. The whiskers extend to Q1 − 1.5× IQR and Q2 + 1.5× IQR. (F) Sensitivities of HCC detection across different tumor stages at varying specificity thresholds.

There is a reduction in the relative amount of fragments with sizes around the 1^st^ peak in the HCC group compared to the HBV and control groups (Figure 4A). The differences in size frequencies between the patient with cancer with the highest tumor DNA fraction (40%) and the median size profile of healthy control samples could be broadly classified into three groups (Figure 4B). Group A (light blue in Figure 4B) exhibited a decrease in size frequency within the range of 42–70 nt. Group B (light green in Figure 4B), within the size range of 70–166 nt, showed an increased signal, followed by a subsequent decrease for sizes greater than 166 nt (group C, light red in Figure 4B). We compared size distributions between representative cancer and healthy control samples to identify characteristic size shifts that might be deemed as cancer-associated patterns. Hence, it would be useful to make use of the information concerning size ranges when analyzing patterns of end motifs. The generally consistent differences in size frequencies were also observed in the pooled samples from HCC and control groups (Figure S4E).

To further explore the diagnostic potential of the fragmentomics markers identified in this work, we developed a support vector machine (SVM) model incorporating all of these markers. This approach was designed to leverage the unique characteristics of the 256 4-mer end motifs derived from PREM, EM5, EM3, and POEM across three distinct size ranges, reflecting the impact of potential size changes between the HCC and non-HCC groups. Each group of the plasma DNA population contributed 256 4-mer end motifs from each of PREM, EM5, EM3, and POEM. Hence, a total of 3,072 features (4 motifs × 256 end motifs × 3 size ranges) could be utilized by the SVM for cancer detection. We adopted a leave-one-out strategy to assess the diagnostic performance (see details in STAR Methods). Receiver operating characteristics (ROC) analysis revealed that EM5 derived from all fragments resulted in an AUC of 0.90. Furthermore, as we gradually included more fragmentomics markers across the three size ranges, the AUC continued to improve, ranging from 0.93 to 0.95 (Figure 4C). The combined analysis of size-stratified end motifs enabled a significant enhancement in cancer detection compared with the conventional EM5 analysis (*p* = 0.01, Delong’s test) (Figure 4D). Using the combined features, the probability of having cancer was significantly higher in patients with HCC compared to CTRs and carriers of HBV (median, 0.938 versus 0.053; range, 0.108–1.00 versus 0.000964–0.886; *p* < 0.0001, two-sided Mann-Whitney *U* test) (Figure 4E). We examined the sensitivity of HCC detection by varying the thresholds of specificity and found that the detection rates of HCC were 86%, 91%, and 93% at specificities of 90%, 85%, and 80%, respectively (Figure 4F). These findings highlight the diagnostic potential of comprehensively integrating the fragmentomics markers identified in this study. In addition, we studied the performance using 1-, 2-, 3-, 4-, and 5-mer end motifs, respectively. As a result, the 4-end motifs gave rise to the best performance (AUC, 0.954) compared with other k-mer features (AUC range, 0.860–0.951) (Figure S5A).

To further validate the robustness of the model, we conducted a bootstrap analysis with 1,000 runs of resampling to assess the 95% confidence interval (CI) of AUC. We compared a combined feature set including EM5, EM3, PREM, and POEM with the conventional EM5-based method for 2-end sequencing. As shown in Figure S5B, the combined feature set achieved an AUC of 0.95 (95% CI, 0.94–0.97) for distinguishing patients with and without HCC, which was significantly higher than the EM5-only model (AUC, 0.90; 95% CI, 0.86–0.93; *p* < 0.01, bootstrap-based test). At a specificity of 95%, the sensitivities were 0.70 (95% CI, 0.61–0.79) for the combined model and 0.58 (95% CI, 0.45–0.76) for the EM5-only model. Figure S5C shows the area under the precision-recall curve (AUPR), where the combined feature set again outperformed EM5 alone (AUPR, 0.93 versus 0.84; 95% CI, 0.91–0.95 versus 0.80–0.90; *p* < 0.01, bootstrap-based test). These bootstrap results support the robustness of the model performance, alleviating concerns regarding potential overfitting.

We noted that the non-HCC group (median, 63 years; range, 26–72) tended to be slightly younger compared with the HCC group (median, 68 years; range, 44–82) (*p* < 0.001, two-sided Mann-Whitney *U* test). We repeated the classification analysis between the age-matched groups (Figures S6A and S6B; see details in STAR Methods). The performance of HCC detection in the age-matched cohort was comparable with that reported in the originally submitted manuscript (AUC, 0.942 versus 0.954; *p* = 0.733, bootstrap-based test) (Figure S6C), suggesting that age is unlikely to be a significant confounder in our study.

### Fragmentomics-based methylation analysis of 3′ ends

Our group previously demonstrated the feasibility of using cfDNA fragmentation patterns to deduce DNA methylation and perform cancer detection, termed fragmentomics-based methylation analysis (FRAGMA).^35^ However, the previously published FRAGMA technology focused on the 5′ ends of cfDNA fragments (i.e., 5′ FRAGMA). We wondered whether the correlation between fragmentation patterns and DNA methylation would also exist in the 3′ ends of cfDNA (3′ FRAGMA).

**Figure 5:**
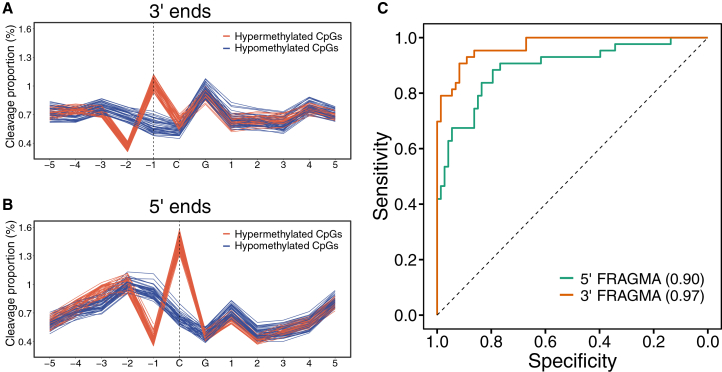
Cleavage proportion of 3′ ends depending on CpG methylation states (A) Cleavage profiles of 3′ ends surrounding the hypermethylated (red lines) and hypomethylated (blue lines) CpGs in plasma DNA of the control group (*n* = 38). (B) Cleavage profiles of 5′ ends surrounding the hypermethylated (red lines) and hypomethylated (blue lines) CpGs. (C) ROC curve analysis of fragmentomics-based methylation analysis at 5′ ends (5′ FRAGMA) and 3′ ends (3′ FRAGMA).

To this end, we adapted a similar analytical strategy for 5′ FRAGMA to analyze 3′ FRAGMA. We determined the cleavage proportion of 3′ ends for each position within a cleavage measurement window as previously described.^35^ As shown in Figure 5A, the 3′ cleavage patterns associated with hypermethylated CpG sites significantly differed from those associated with hypomethylated CpG sites in healthy controls. For example, the positions −4 and −1 exhibited higher cleavage proportion values in the population of cfDNA molecules associated with hypermethylated CpG sites (median, 0.75 and 1.03) compared with those associated with hypomethylated CpG sites (median, 0.70 and 0.57). The 3′ ends were most frequently terminated at the position 1 nt immediately before a methylated CpG site. In contrast, for 5′ ends, the positions at cytosines of CpG sites exhibited higher cleavage proportion values in the population of cfDNA molecules associated with hypermethylated CpG sites than those associated with hypomethylated CpG sites (median cleavage proportion, 1.45 versus 0.64) (Figure 5B). The 5′ ends preferred the position exactly at a cytosine of a methylated CpG site, which was in line with the previous finding.^35^

To evaluate the diagnostic performance of using 3′ FRAGMA, we employed an SVM to analyze 3′ cleavage ends associated with differentially methylated CpG sites to distinguish patients with and without HCC (see details in STAR Methods). The probability of having cancer was significantly higher in patients with HCC compared to CTRs and carriers of HBV (median, 0.98 versus 0.02; interquartile range [IQR], 0.80–1.00 versus 0.003–0.17; *p* < 0.001, two-sided Mann-Whitney *U* test) (Figure S7). This finding suggests that 3′ FRAGMA could be used to measure DNA methylation and to detect cancer . Notably, 3′ FRAGMA demonstrated superior performance to 5′ FRAGMA, with the AUC increasing to 0.97 from 0.90 (*p* < 0.01, DeLong’s test) (Figure 5C). Using a cutoff of 0.32 for the probability of having cancer, the specificity and sensitivity were 0.91 and 0.90, respectively. At specificity thresholds of 85% and 80%, the corresponding sensitivities were both 95%.

### Holistic determination of the four ends of a double-stranded cfDNA molecule

We have demonstrated that the inclusion of 3′ fragmentomics markers of cfDNA is beneficial for cancer detection. We next explored whether it might be possible to holistically determine all four ends of a double-stranded cfDNA molecule and to use fragmentomics information at all four ends for cancer diagnostics. However, the nature of 2-end sequencing makes it challenging to trace all ends of a double-stranded molecule from sequencing results, as the original double-stranded cfDNA molecules have been separated into single strands prior to sequencing. To this end, we developed a novel sequencing approach to solve this issue.

As shown in Figure S8, double-stranded cfDNA molecules exhibit various end modalities, such as 3′ protruding ends, 5′ protruding ends, or blunt ends. These cfDNA molecules are ligated with stem-loop adapters, featuring customized end configurations capable of hybridizing to the native cfDNA molecules of interest. For instance, a cfDNA fragment having a 5′ protruding strand would be ligated with a matched stem-loop adapter that has the exact length of single-stranded sequence complementary to the protruding end of that molecule. The end information, including length and base composition, was encoded in the sequence barcode within the stem region of the adapter (see details in STAR Methods). Therefore, properly ligated molecules formed closed circular DNA. An additional step involving exonuclease treatment was implemented to eliminate incompletely ligated products, minimizing the risk of misreading barcode information caused by erroneous ligations. The circularized DNA molecules were then sequenced using single-molecule real-time sequencing (SMRT-seq; Pacific Biosciences [PacBio]). In this method, both strands of the same molecule are sequenced multiple times, enabling the decoding of the complete 4-end information for a single double-stranded molecule (see details in STAR Methods). We shall refer to this methodology as 4-end sequencing.

Using 4-end sequencing, we analyzed samples from 6 CTRs, 6 carriers of HBV, and 10 patients with HCC, with a median of 75,706 molecules (IQR, 46,377–166,786). We observed that the median frequencies of PREM, EM5, EM3, and POEM were generally correlated with those analyzed by 2-end sequencing (Figures S9A–S9D), and the top 20 motifs largely overlapped for each category of end motif, with a mean of 65% shared between the two technologies (Table S3). However, EM3 appeared to be less correlated compared to other types of end motifs. The observed discrepancy between 2-end and 4-end sequencing might stem from inherent differences in the technologies. For example, 2-end sequencing measured both ssDNA and dsDNA molecules, whereas 4-end sequencing could only detect dsDNA molecules. Each type of end motif demonstrated a certain degree of power in differentiating between HCC and non-HCC groups according to the SVM analyses, with AUC values ranging from 0.90 to 0.96 (Figure S9E).

**Figure 6:**
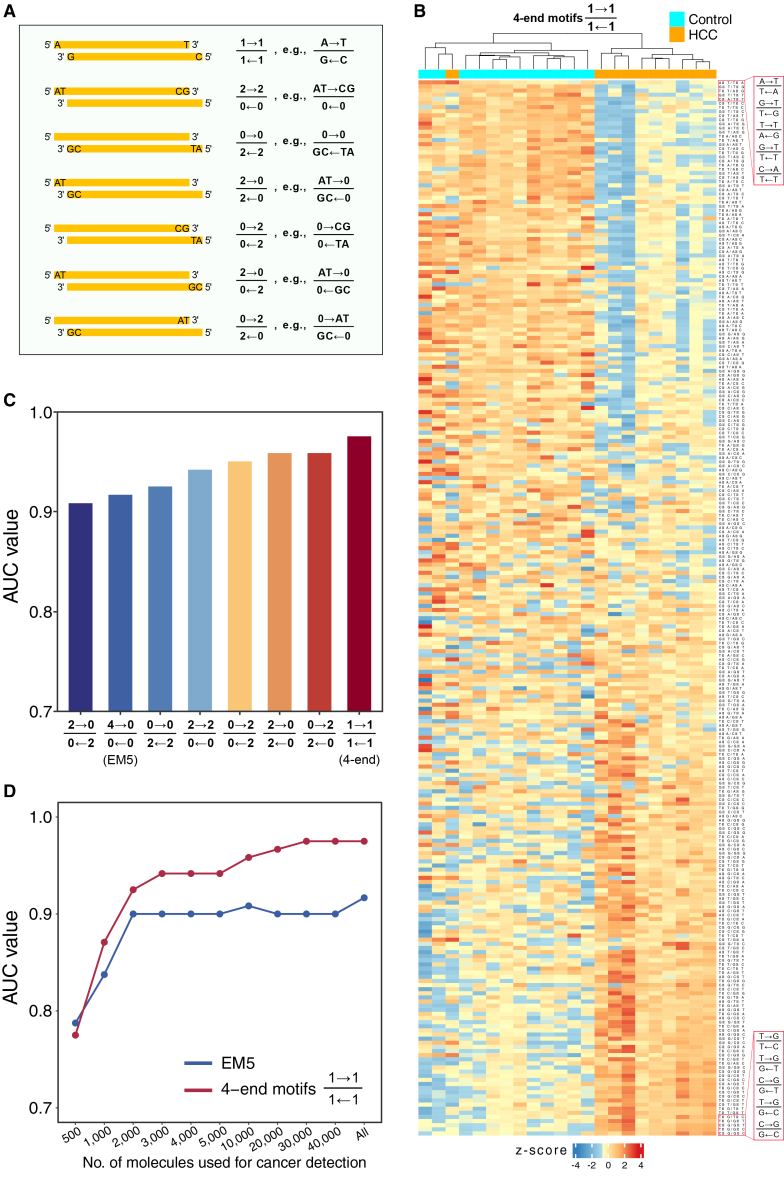
Nomenclature and application of 4-end sequencing technology (A) The terminologies of novel 4-mer end motifs deduced from 4-end sequencing. Representative 4-mer end motifs include $\frac{1\to 1}{1\leftarrow 1}$, $\frac{2\to 2}{0\leftarrow 0}$, $\frac{0\to 0}{2\leftarrow 2}$, $\frac{2\to 0}{2\leftarrow 0}$, $\frac{0\to 2}{0\leftarrow 2}\text{,}$$\frac{2\to 0}{0\leftarrow 2}$, and $\frac{0\to 2}{2\leftarrow 0}$. In this notation, the two ends of the Watson strand in a 5′-to-3′ direction, as indicated by the arrow, are placed in the numerator, and the two ends of the Crick strand are placed in the denominator. The number denotes the motif length from an end (e.g., a value of 1 corresponds to a 1-mer motif). For example, $\frac{1\to 1}{1\leftarrow 1}$ indicates that the Watson and Crick strands of a dsDNA each contribute two 2 ends to this motif, referred to as a 4-end motif. (B) Heatmap analysis of 256 motif frequencies in 4-end motif $\frac{1\to 1}{1\leftarrow 1}$ in the plasma DNA fragments between non-HCC (*n* = 12) and HCC (*n* = 10) groups. (C) AUC values of $\frac{2\to 0}{0\leftarrow 2}$, $\frac{4\to 0}{0\leftarrow 0}$, $\frac{0\to 0}{2\leftarrow 2}$, $\frac{2\to 2}{0\leftarrow 0}$, $\frac{0\to 2}{0\leftarrow 2}\text{,}$$\frac{2\to 0}{2\leftarrow 0}$, $\frac{0\to 2}{2\leftarrow 0}$, and $\frac{1\to 1}{1\leftarrow 1}$ motifs in differentiating patients with HCC from those without. (D) Performance of 4-end motif and EM5 analyses in HCC detection using the downsampling analysis of sequenced fragments, including 500, 1,000, 2,000, 3,000, 4,000, 5,000, 10,000, 20,000, 30,000, and 40,000 fragments.

The limited number of sequenced fragments available from SMRT-seq represented challenges faced by this first implementation of the 4-end sequencing concept. Hence, as a proof-of-concept study, instead of studying 4-mer end motifs for each of the 4 ends, we studied only a single-nucleotide end motif from each end. We also developed a specific notation for such 4-end fragmentomics analyses. For the analysis of 1-mer motif at each of the 4 ends, we will denote this as $\frac{1\to 1}{1\leftarrow 1}$. In this notation, the two ends of the Watson strand in a 5′-to-3′ direction are placed in the numerator, and the direction is indicated by the arrow. The two ends of the Crick strand are placed in the denominator. The number denotes the length of a motif from an end (e.g., a value of 1 corresponds to a 1-mer motif). Using this notation, analysis of the 2-mer motif at each of the 5′ and 3′ ends of one strand will be denoted as $\frac{2\to 2}{0\leftarrow 0}$ or $\frac{0\to 0}{2\leftarrow 2}$. Examples of the use of this notation are shown in Figure 6A. In certain applications, it might be beneficial to show the actual sequence of a particular motif instead of just its length. Hence, for these applications, the actual nucleotide sequence could be stated explicitly (see the right-hand side of Figure 6A). For example, assuming that the terminal 5′-AT-3′ dinucleotide is present in EM3 of the Watson strand and the terminal 5′-CG-3′ dinucleotide is in EM3 of the Crick strand, the 4-end motif could be denoted as $\frac{0\to \text{AT}}{\text{GC}\leftarrow 0}$.

Hierarchical clustering analysis of the frequencies of the 256 4-end motifs revealed that subjects with HCC clearly clustered together, while non-HCC subjects formed the other distinct clusters (Figure 6B). Compared with the control group, the top 5 most decreased 4-end motifs in the HCC group were $\frac{A\to T}{T\leftarrow A}$, $\frac{G\to T}{T\leftarrow G}$, $\frac{T\to T}{A\leftarrow G}$, $\frac{G\to T}{T\leftarrow T}$, and $\frac{C\to A}{T\leftarrow T}$. Conversely, the top 5 most increased 4-end motifs in the HCC group were $\frac{T\to G}{T\leftarrow C}$, $\frac{T\to G}{G\leftarrow T}$, $\frac{C\to G}{G\leftarrow T}$, $\frac{T\to G}{G\leftarrow C}$, and $\frac{C\to G}{G\leftarrow C}$. Furthermore, we utilized an SVM to analyze the classification power of the different types of end motifs defined in the 4-end sequencing results. The use of 4-end motifs resulted in the best performance for differentiating patients with HCC from those without, achieving an AUC of 0.98, while the other types of end motifs had AUC values ranging from 0.91 to 0.96 (Figure 6C).

To further investigate how the number of sequenced fragments would affect the performance of the 4-end sequencing for cancer detection, we carried out downsampling analysis of sequenced fragments by randomly selecting 500, 1,000, 2,000, 3,000, 4,000, 5,000, 10,000, 20,000, 30,000, and 40,000 fragments for classification analysis. Figure 6D shows that the performance of 4-end-motif analysis progressively improved as the number of sequenced fragments increased. For example, with the use of 1,000 sequenced fragments, the AUC was 0.87, whereas the AUC increased to 0.96 with 10,000 sequenced fragments. The plateau of the performance was reached at 20,000 sequenced fragments. Notably, the use of 4-end-motif analysis showed superior performance to EM5 analysis for distinguishing between patients with and without HCC across the different numbers of sequence fragments being analyzed. Even using 4 times more sequence fragments than the amount required to achieve an AUC of 0.95 with 4-end motifs, the EM5 analysis still failed to reach the same level of performance. The data suggest that the holistic analysis of all 4 termini of a double-stranded cfDNA molecule offers a synergistic enhancement in diagnostic performance.

### Biological insights into coordinated fragmentation of cfDNA by nucleases using 4-end sequencing

**Figure 7:**
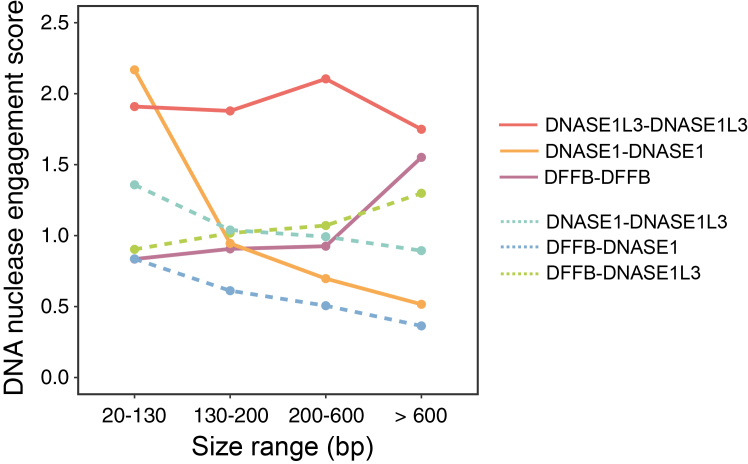
Dynamic engagement of DNASE1L3, DNASE1, or DFFB across different cfDNA populations concerning different size ranges deduced from 4-end sequencing

The role of nucleases in cfDNA fragmentation has been revealed using nuclease-knockout mice and *in vitro* whole-blood incubation assays.^14^^,^^17^^,^^36^ However, it remains elusive whether multiple DNA nucleases in plasma act in a coordinated manner during cfDNA fragmentation. To address this question, we used 4-end sequencing to study the engagement of DNA nucleases across different populations of cfDNA molecules in terms of fragment sizes. We defined the nuclease-specific 5′ end motifs and 3′ end motifs as nuclease-cutting signatures, according to the differential motif frequencies between wild-type and nuclease-knockout mice (see STAR Methods). We analyzed DNASE1L3-, DNASE1-, and DFFB-cutting end signatures at both the 5′ and 3′ ends on the same side of each fragment (i.e., $\frac{2\to 0}{2\leftarrow 0}$ or $\frac{0\to 2}{0\leftarrow 2}$ ), forming DNASE1L3-DNASE1L3 (i.e., $\frac{\text{DNASE}1L3\to 0}{\text{DNASE}1L3\leftarrow 0}\;or\;\frac{0\to \text{DNASE}1L3}{0\leftarrow \text{DNASE}1L3}$), DNASE1-DNASE1 (i.e., $\frac{\text{DNASE}1\to 0}{\text{DNASE}1\leftarrow 0}$ or $\frac{0\to \text{DNASE}1}{0\leftarrow \text{DNASE}1}$), and DFFB-DFFB (i.e., $\frac{\text{DFFB}\to 0}{\text{DFFB}\leftarrow 0}\;\text{or}\;\frac{0\to \text{DFFB}}{0\leftarrow \text{DFFB}})$ signatures, respectively. cfDNA fragments, mainly ranging from 130 to 200 bp, were characterized by DNASE1L3-DNASE1L3 signatures, supporting the role of DNASE1L3 as a major contributor to cfDNA fragmentation (Figure 7). Interestingly, the abundance of DFFB-DFFB signatures increased with fragment sizes, particularly for those fragments >600 bp, suggesting a role for DFFB in the early stages of cfDNA fragmentation. In contrast, the amount of DNASE1-DNASE1 signatures was more prevalent in shorter cfDNA fragments (size range, 20–130 bp) and diminished in longer ones, indicating that DNASE1 might be involved in downstream fragmentation events. The DNASE1L3-DNASE1L3 signature remained consistently substantial across a broad range of fragment sizes, implying that DNASE1L3 might participate in both early and late stages of fragmentation. We also observed mixed cleavage patterns, where the 5′ and 3′ ends on the same side of a cfDNA fragment were associated with different nucleases, such as DNASE1-DNASE1L3, DFFB-DNASE1, and DFFB-DNASE1L3. In the short molecules (<130 bp), the homogenous cleavage patterns (e.g., DNASE1L3-DNASE1L3 and DNASE1-DNASE1) were found to be more frequent than the heterogeneous cleavage patterns (e.g., DNASE1-DNASE1L3). Similarly, in the long molecules (>600 bp), homogenous cleavage patterns, such as DNASE1L3-DNASE1L3 and DFFB-DFFB, were more common than mixed signatures, such as DFFB-DNASE1L3. These findings suggest that once a DNA nuclease initiates a cleavage on one strand, it might also coordinate the subsequent proximal cleavage on the complementary strand. These findings demonstrated the power of 4-end sequencing for dissecting the dynamic and coordinated roles of multiple DNA nucleases in cfDNA fragmentation, offering new insights into the complexity of this biological process. Of note, the 1^st^ peak observed in the single-stranded cfDNA libraries did not appear in the size distribution of 4-end sequencing that profiled the double-stranded moleucles (Figure S9F). Due to the lower throughput of this platform, we expanded the size ranges to ensure sufficient data for end-motif analysis. The size stratification used in this context differed from that employed in the single-stranded cfDNA analysis.

Another feature of 4-end sequencing allows for assessing both the 5′ and 3′ jagged ends of cfDNA resulting from the coordinated cleavages. Of note, the 3′ jagged ends could not be analyzed in the previously published method.^20^ In this study, we found that the amount of fragments with 3′ jagged ends was significantly reduced in *Dnase1l3*^−/−^ mice (median: 23.28% and 9.79%). Knocking out DFFB led to the reduction of blunt ends, and the deletion of DNASE1 tended to decrease 5′ jagged ends (Figure S10).

## Discussion

In this study, we have made significant strides in the holistic analysis of native termini of cfDNA fragments. ssDNA library preparation allows the direct ligation of sequencing adapters to denatured ssDNA molecules. This method facilitates the determination of both ends of an individual DNA strand, known as 2-end sequencing. The end motifs identified from 2-end sequencing, such as PREM, EM3, and POEM, exhibit unique biological properties and add extra dimensions to cancer detection. For example, the integrated analysis of these four types of end motifs significantly improved the diagnostic power for HCC detection. Notably, the 3′ ends were also found to correlate with cfDNA methylation (3′ FRAGMA), in addition to the previously reported 5′ ends (5′ FRAGMA). Compared with 5′ FRAGMA, the use of 3′ FRAGMA signals has enhanced cancer detection, achieving an AUC of 0.97, up from 0.90. Motivated by the added diagnostic value of 3′ ends, we further developed a new approach for the holistic determination of all four ends of a double-stranded cfDNA molecule, referred to as 4-end sequencing. The findings of 4-end sequencing suggest the synergistic effect in cancer detection when using all four termini of plasma DNA, opening up many exciting possibilities for developing diagnostic tools of liquid biopsy.

Given that DNA nuclease-mediated cleavages of cfDNA produce both 5′ and 3′ ends simultaneously, one might initially expect PREM to be identical to EM3 and POEM to be identical to EM5. However, upon examining their actual patterns, we observed that they each exhibited distinct motif patterns, while PREM and EM3 showed a general correlation in motif frequencies. A similar observation was made for the relationship between POEM and EM5. One plausible explanation for these discrepancies might be the involvement of additional DNA degradation processes following the initial nuclease-mediated cleavage events. For instance, exonuclease activity might further trim the DNA ends, or secondary endonuclease cleavages occurring near the initial cut sites could also contribute to the divergence observed between these paired-end motifs. Interestingly, the similarity between PREM and EM3, as well as between POEM and EM5, was significantly enhanced for cfDNA within a specific size range of 146–186 nt, compared to another size range of 32–72 nt. This suggests that longer cfDNA fragments may have been subjected to a lesser degree of degradation than shorter cfDNA fragments.

Harkins et al. attempted to study the native ends of fragmented cfDNA molecules, addressing the information loss inherent in traditional library preparation,^37^ but did not enable the concurrent analysis of all ends from a DNA molecule for the following reason. Their approach utilized a two-step ligation process to covalently tag double-stranded sequencing adapters (i.e., P5 and P7 strands) with a unique end identifier (UEI) to the DNA molecules of interest, followed by sequencing on the Illumina platform. The UEI was a barcode sequence indicating the length and identity (5′ or 3′) of the overhang. However, as noted in their publication,^37^ the P5 strand could be ligated to the ends during the first step, regardless of whether the UEI matched the desired end modalities of the DNA substrate or not, thus introducing incorrect ligation products. The second step involving the P7 strand ligation depended on the accurate ligation of the first P5 strand. Therefore, only the ends related to P7 in the sequenced result might accurately reflect the original cfDNA termini and were ultimately considered valid in Harkins et al.’s study, whereas the other ends related to P5 were error-prone and discarded,^37^ thus hindering the decoding of all ends in a molecule.

In contrast, our work introduced two different methods for simultaneously and accurately interrogating native ends in the sequenced results, namely, 2-end and 4-end sequencing. 2-end sequencing adapted ssDNA library preparation and assessed ends from the two strands separately using short-read sequencing (Illumina), whereas 4-end sequencing enabled the concurrent assessment of all ends from both strands using long-read sequencing (PacBio). Importantly, for 4-end sequencing, we use exonucleases to remove incomplete ligation products prior to sequencing. Additionally, SMRT-seq selectively sequences completely ligated circular DNA molecules, as the DNA polymerase can only perform multiple laps of continuous and processive polymerization on the intact circularized DNA molecules during sequencing. Therefore, such a two-step selection ensures that only dsDNA molecules that are properly circularized are analyzed by 4-end sequencing, providing high-fidelity end information of each single dsDNA molecule.

Guo et al. analyzed the breakpoint motifs of plasma DNA for lung cancer detection.^38^ The breakpoint motifs included the nucleotides surrounding the 5′ end of a plasma DNA fragment. Thus, the end information related to 3′ ends has not been studied and was not utilized in that study. Budhraja et al. used the correlations of base frequencies for positions surrounding the 5′ fragment ends and the information-weighted fraction of aberrant fragments to train a random forest classifier for cancer detection.^39^^,^^40^ Of note, Budhraja et al. analyzed the frequencies of bases flanking 5′ cleavage sites individually but did not concurrently consider physical linkage among these individual bases.^39^

It is noteworthy that 4-end sequencing offers a more comprehensive determination of cfDNA ends, adding to the diagnostic value of cfDNA. For example, in the detection of patients with HCC, the diagnostic performance using all ends from both strands of a dsDNA molecule ($\frac{1\to 1}{1\leftarrow 1}$) was consistently superior to other end-motif combinations, such as conventional 5′ 4-mer end motifs (EM5). On the other hand, this technology makes it possible to trace the dynamic activity of multiple DNA nucleases involved in the cfDNA fragmentation process. Such capability may pave the way for deeper investigations into the interplay of nuclease activities in plasma in other diseases, such as systemic lupus erythematosus. However, the throughput of the PacBio Sequel II platform used in the current study was suboptimal, limiting the full realization of 4-end sequencing’s potential. The increasing throughput of newer PacBio platforms, such as the Revio system, holds promise for expanding the applicability of 4-end sequencing to broader scientific and clinical research and eventual clinical applications. Furthermore, the adaptation of 4-end sequencing for compatibility with the Illumina platform is being pursued by ongoing work in our group. On the other hand, we conjecture that the 2-end sequencing approach can be readily adapted to other sequencing-by-synthesis platforms, such as Element, Ultima, and MGI, via library conversion. ONT libraries can also be built on top of 2-end libraries with dedicated adapter trimming required for end-motif analysis. In contrast, the current implementation of the 4-end approach, as reported here, relies on circularized molecules specific to PacBio and is not compatible with platforms requiring linear DNA.

Accumulating evidence indicates that integrating various established features of plasma DNA into a combinatory model could be highly beneficial.^16^^,^^41^^,^^42^ These features include fragment sizes, 5′ end motifs, cfDNA coverages, copy-number aberrations, mutations, and methylation patterns. Such integration has shown promise in improving the detection of cancers, such as lung cancer, colorectal cancer, pancreatic cancer, and nasopharyngeal carcinoma, in several recently published large-scale cohort studies.^10^^,^^16^^,^^42^^,^^43^ The end signatures identified in this study would add additional dimensions to the combinatory model, potentially enhancing its diagnostic performance even further.

In summary, this study presents a comprehensive characterization of cfDNA ends, allowing the development of previously unrecognized classes of fragmentomics markers, with diagnostic applications in cancer liquid biopsy. The holistic determination of cfDNA ends offers a powerful means to unveil new biological properties and scrutinize subtle “cancer signals.” Such technological developments also have applications in other types of liquid biopsies, e.g., for prenatal testing and transplantation monitoring.

### Limitations of the study

This proof-of-concept study potentially has several limitations. First, the clinical sample size was relatively small, particularly for the 4-end sequencing dataset, highlighting the need for larger cohorts to validate these newly identified fragmentomics markers. Second, sequencing throughput was limited (<1 million reads per sample) due to its reliance on the PacBio platform. To address this, we are undertaking further research to increase data yield and enable more robust downstream analyses. Finally, in this study, only an SVM was employed to demonstrate the synergistic use of a constellation of multiple newly identified fragmentomics markers. With expanded sample sizes and higher-throughput sequencing data, future work will incorporate more sophisticated model architectures for further enhancing diagnostic performance.

## Resource availability

### Lead contact

Further information and requests for resources and reagents should be directed to and will be fulfilled by the lead contact, Y.M. Dennis Lo (loym@cuhk.edu.hk).

### Materials availability

The adapters used in 4-end sequencing with the PacBio platform are newly developed reagents, and their sequences are listed in Table S5.

### Data and code availability

Raw sequencing data used to generate results reported in the manuscript are stored in the European Genome-phenome Archive (EGA) with the project no. EGAS00001003409 (https://ega-archive.org/studies/EGAS00001003409). Computer codes for performing fragmentomics analysis have been deposited in Zenodo with the repository link (https://doi.org/10.5281/zenodo.18150435).

## Acknowledgments

This work was supported by the Innovation and Technology Fund under the InnoHK Initiative, a major initiative of the Hong Kong Special Administrative Region Government, and the State Key Laboratory of Translation Oncology, funded by the Innovation and Technology Commission. Y.M.D.L. is supported by an endowed chair from the Li Ka Shing Foundation. We thank Suk Hang Cheng, Man Na Law, and Laura Simone Elisabeth Schulz for their technical assistance.

## Author contributions

P.J., M.-J.L.M., R.Q., K.C.A.C., and Y.M.D.L. designed the study. W.K.J.L., S.C.Y.Y., L.Y.L.C., G.L.H.W., L.L.C., J.W., S.L.C., and V.W.S.W. performed case recruitment and analyzed the clinical data. M.-J.L.M., Y.S., J.L., Q.Z., W.H.A.T., Y.M., and S.L.C. performed the bench work and the sequencing experiments. R.Q., W.P., J.B., G.K., and D.X. performed the bioinformatics analyses. P.J., M.-J.L.M., R.Q., Y.S., J.L., K.C.A.C., and Y.M.D.L. performed data analysis and wrote the manuscript. All authors contributed to the revision of the manuscript and approved the final version. Y.M.D.L. obtained funding and supervised the study.

## Declaration of interests

Y.M.D.L. holds equities in DRA, Take2, and Insighta. K.C.A.C. holds equities in DRA, Take2, Insighta, and Illumina. P.J. and W.K.J.L. hold equities in Illumina. P.J. is a consultant to Take2. P.J. is a director of DRA and KingMed Future. W.K.J.L. is a director of DRA. Patent royalties are received from Grail, Illumina, LabCorp, DRA, Take2, Insighta, and Xcelom. G.L.H.W. serves as an advisory committee member for AstraZeneca, Barinthus Biotherapeutics, Gilead Sciences, GlaxoSmithKline, Janssen, and Virion Therapeutics. G.L.H.W. delivered invited talks as a speaker for Abbott, AbbVie, Ascletis, Bristol-Myers Squibb, Echosens, Ferring, Gilead Sciences, Janssen, and Roche. G.L.H.W. has received research funding from Gilead Sciences. P.J., M.-J.L.M., R.Q., D.X., K.C.A.C., and Y.M.D.L. have filed a patent application based on the data presented in this study (patent application no. 19/282,996).

## STAR★Methods

### Key resources table

**Table:** 

REAGENT or RESOURCE	SOURCE	IDENTIFIER
**Biological samples**		

Human plasma from healthy controls	The Chinese University of Hong Kong	N/A
Human plasma from patients with chronic hepatitis B	The Chinese University of Hong Kong	N/A
Human plasma from patients with hepatocellular carcinoma	The Chinese University of Hong Kong	N/A
Plasma from mice with genotypes *Dnase1l3*^−/−^	The Chinese University of Hong Kong	N/A
Plasma from mice with genotypes *Dnase1*^−/−^	The Chinese University of Hong Kong	N/A
Plasma from mice with genotypes *Dffb*^−/−^	The Chinese University of Hong Kong	N/A
Plasma from wild-type mice	The Chinese University of Hong Kong	N/A

**Critical commercial assays**		

EZ1&2 ccfDNA Kit	Qiagen	954854
EZ2 Connect	Qiagen	9003210
QIAamp® Circulating Nucleic Acid Kit	Qiagen	55114
QIAamp® DNA Blood Mini Kit	Qiagen	51104
SRSLY® PicoPlus DNA NGS Library Preparation Base Kit	Claret Bioscience	CBS-K250B-96
UMI Addition Bundle 96 UMI-UDI Primer Plates for 96 Reactions	Claret Bioscience	CBS-UMB-96
T4 Polynucleotide Kinase	New England Biolabs	M0202L
T4 DNA ligase buffer	New England Biolabs	B0202S
T4 DNA ligase	New England Biolabs	M0202L
SMRTbell® enzyme cleanup kit 2.0	Pacific Biosciences	101-932-600
Qubit™ 1× dsDNA High Sensitivity (HS) Assay Kits	ThermoFisher	Q33231
TruSeq Nano DNA Library Prep Kit	Illumina	20015964
AMPure XP beads	Beckman Coulter	A63881

**Deposited data**		

Raw sequencing data	this study	EGA: EGAS00001003409
Fragmentomics analysis code	this study	Zenodo: https://doi.org/10.5281/zenodo.18150435

**Software andalgorithms**		

SOAP2	Li et al.^44^	https://github.com/gigascience/bgi-soap2
R4.3.1	Ihaka and Gentleman^45^	https://cran.r-project.org
Python3.8.8	van Rossum and de Boer^46^	https://www.python.org/downloads/

**Other**		

PacBio (Sequel II)	PacBio	https://www.pacb.com/technology/hifi-sequencing/sequel-system/
Illumina NovaSeq 6000	Illumina	https://www.illumina.com/

### Experimental model and study participant details

Human studies were approved by the Joint Chinese University of Hong Kong – Hospital Authority New Territories East Cluster Clinical Research Ethics Committee. Healthy control subjects (*n* = 44), patients with chronic hepatitis B but without HCC (*n* = 41), and patients with HCC (*n* = 53) were recruited with informed written consent from the Prince of Wales Hospital, Hong Kong. We recorded demographic parameters at the time of sample recruitment, such as sex, age, smoking status, and anti-HBV medication for individuals in this cohort. As shown in Table S4, there was no statistically significant difference between non-cancer and cancer groups with respect to sex (*p* = 0.6, Fisher’s exact test) or smoking status (*p* = 0.1, Fisher’s exact test). Of note, the healthy control group (median, 64 years; range, 26–72) and the HBV carrier group (median, 62 years; range, 45–72) tended to be slightly younger, compared with the HCC group (median, 68 years; range, 44–82) (*p* < 0.001, two-sided Mann-Whitney *U* test). The healthy control group and the HBV carrier group were not statistically significantly different (*p* = 0.91, two-sided Mann-Whitney *U* test).

Animal studies were approved by the Animal Experimentation Ethics Committee of The Chinese University of Hong Kong. Mice with genotypes *Dnase1l3*^−/−^ (*n* = 11), *Dnase1*^−/−^ (*n* = 10), *Dffb*^−/−^ (*n* = 9), and wild-type mice (*n* = 20) on C57BL/6 genetic background were analyzed.

### Method details

#### Sample collection

Peripheral blood samples were collected from study participants or animal models using EDTA blood tubes and subsequently processed according to the published protocols.^13^^,^^17^ In brief, the peripheral blood samples were first centrifuged at 1,600 × g for 10 min at 4°C. After the centrifugation, the plasma portion was further centrifuged at 16,000 × g for 10 min at 4°C to pellet the residual cells and platelets. The plasma samples were harvested and stored at −80°C until further use.

#### DNA extraction and library preparation for 2-end sequencing

Plasma DNA was extracted from 2 mL of plasma using the EZ1&2 ccfDNA Kit (QIAGEN) that was compatible with the automation equipment EZ2 Connect (QIAGEN). DNA library preparation was constructed using the SRSLY PicoPlus DNA NGS Library Preparation Base Kit with the UMI-UDI Primer Set (Claret Bioscience) according to the manufacturer’s instructions. In brief, plasma DNA containing both dsDNA and ssDNA was denatured into ssDNA molecules and subsequently ligated with SRSLY splint adapters. Each SRSLY splint adapter contains a 7-nt random single-stranded overhang, allowing the complementary pairing between SRSLY splint adapters and ending sequences of ssDNA molecules. The adapter-ligated molecules were subsequently amplified through PCR, during which unique molecular identifiers (UMIs) and sample-specific indexes were incorporated. As there was no end-repairing step, the native ends of cfDNA fragments could be retained. The libraries were sequenced on the NovaSeq 6000 system (Illumina) in a 100-bp × 2 paired-end mode.

As shown in Figure S11, the 2-end sequencing involves paired-end reads sequenced in the 5′–3′ direction. The design of the sequencing primer makes the first sequencing cycle correspond to the first base of the insert DNA. Adapter sequences may only be present at the 3′ ends of read 1 or read 2. Importantly, the 5′ ends of read 1 and read 2 reflect the native 5′ and 3′ termini of the original cfDNA fragments, respectively. Therefore, trimming the 3′ ends does not compromise the integrity of the native termini captured by this method.

#### DNA extraction and library preparation for 4-end sequencing

Plasma DNA was extracted from 2 mL of plasma using the QIAamp Circulating Nucleic Acid Kit (Qiagen) according to the manufacturer’s protocol. The following library preparation method was used for 4-end sequencing. DNA was first phosphorylated using 20 units of T4 Polynucleotide Kinase (New England Biolabs, M0201L) and 1× T4 DNA ligase buffer in a 50 μL reaction, which was incubated at 37°C for 30 min. Afterward, 1,200 units of T4 DNA ligase (New England Biolabs, M0202L) and 4 μL of 20 μM pooled stem-loop adapters were added to the reaction mixture and further incubated at 25°C overnight. The customized stem-loop adapters (IDT) consist of three main components: an end typing adapter, a 6-bp barcode, and a PacBio sequencing primer binding site. End typing adapter carries either a blunt end or a single-stranded overhang with various lengths, ranging from 1 to 20 nucleotides. A 6-bp barcode encodes the type of ends (e.g., blunt, 5′ protruding, or 3' protruding end) and the length of the overhang. A total of 41 types of stem-loop adapters were mixed in an equal-molar manner, including 1 blunt end adapter, 20 adapters with 3′ protruding overhangs (ranging from 1 to 20 nucleotides in length), and 20 adapters with 5' protruding overhangs (ranging from 1 to 20 nucleotides in length). Detailed information of these customized adapters was listed in Table S5. After the adapter ligation, the SMRTbell enzyme cleanup kit 2.0 was used to remove any linear molecules caused by either incomplete adapter ligation or DNA damage. Only DNA molecules with complete circularization could be successfully sequenced in a PacBio Sequel II platform using an SMRT Cell 8M flowcell, using a Sequel II Sequencing 2.0 Kit (PacBio).

As shown in Figure S12, the entire double-stranded cfDNA molecule is sequenced, including both the plus and minus strands as well as the adapter regions. The adapters contain barcode sequences that encode critical information such as jagged end types, overhang length and base composition of the adapters. By leveraging this barcode information, we can precisely identify and remove the adapter sequences without compromising the native end motifs of the cfDNA fragments.

#### Sequencing alignment and motif determination

For sequencing data generated from the 2-end sequencing (using the Illumina platform), raw reads were first subjected to a quality control process by which adapter sequences and low-quality bases (i.e., with Phred scores below 20) were removed. The resulting high-quality reads were aligned to the human reference genome (NCBI37/hg19) or mouse reference genome (GRCm38/mm10) using the Sort Oligonucleotide Alignment Program 2 (SOAP2). Only uniquely mapped paired-end reads with no more than two mismatches, whose insert sizes are required to be within a range of 20–600 bp, were retained for downstream analysis. To assess the impact of updated human reference genomes, we aligned raw sequencing reads to hg38 (Genome Reference Consortium Human Build 38), CHM13 (Telomere-to-Telomere CHM13 Assembly), and CN1 (Chinese Pangenome Reference CN1), and calculated 4-mer end motif frequencies for each motif type. Pearson’s r values were 1.0 across all categories (Figure S13), suggesting that the overall impact of reference genome choice on motif analysis might be negligible. This interpretation was further supported by the fact that a model trained on hg19-derived data was generalized well to CHM13-derived data, with classification performance remaining comparable (Figure S14). We noted several motifs slightly deviating from the diagonal line when comparing the motif frequencies using reads aligned to hg19 and CHM13 (Figure S13; Table S6), which were likely attributable to centromeric, telomeric, and large segmental duplication regions^47^ (Figure S15).

For 4-end sequencing (PacBio platform) data, circular consensus sequences (CCSs) were first demultiplexed by removing barcode sequences and subsequently aligned to the hg19 or mm10 reference genome using pbmm2. Only CCS reads, which had high mapping quality scores of >30 and no soft clipping, were used for further analysis.

The first k-nt sequence on each end of plasma DNA molecules was determined as a k-mer end motif, denoted in a 5′ to 3' direction. The frequency of each of the possible motifs was calculated and normalized by the total number of ends. To minimize the bias caused by potential pre-analytical factors (e.g., different experimental protocols), the sequencing results of sonicated DNA by 2-end sequencing and 4-end sequencing were obtained and used to correct the pre-analytical bias by the formula below:

$$Corrected\;F_{i}\;in\;plasma=\frac{Observed\;F_{i}\;in\;plasma\;}{Observed\;F_{i}\;in\;sonicated\;DNA}\times Expected\;F_{i},$$

where for a motif ***i***, its motif frequency is denoted by *F*_*i*_. The observed *F*_*i*_ in plasma and sonicated DNA were obtained from corresponding sequencing results, respectively, and the expected *F*_*i*_was determined *in silico* by counting 4-mer motifs in a reference genome using a sliding window method.

#### Analysis of the differential motifs

To identify differential motifs between HCC and non-HCC groups, a two-sided Wilcoxon rank-sum test was conducted to compare motif frequencies between the HCC and control groups with adjusted *p* values based on the Benjamini-Hochberg method. Motifs with an adjusted *p* < 0.05 were considered statistically significant. For these differential motifs, those with fold changes in median motif frequency between the HCC and non-HCC group >1 were defined as differentially increased motifs in HCC, whereas those with fold changes <1 were considered differentially decreased motifs in HCC.

#### Machine learning model

For one motif type (e.g., EM5), the input features for the support vector machine (SVM)^48^ consisted of 256 motif frequencies calculated from cfDNA molecules at a specific size range (i.e., 256 features). When inputting 4 types of motifs from 3 size ranges to the SVM, the total number of features was 3,072 (i.e., 256 × 4 × 3 features).

Leave-one-out cross-validation (LOOCV) strategy was utilized in the machine learning model. For a dataset containing N samples, one sample was iteratively excluded as the testing sample, while the remaining *N* - 1 samples were used to train the SVM classifier. The trained model was then used to predict whether the left-out sample originated from the cancer group or control group. This process was repeated N times, such that each sample served once as the test case. As a result, a prediction was generated for every sample in the dataset. Classification accuracy was then calculated based on the aggregated predictions across all iterations. There is no feature selection and scaling that is exclusively performed within the training split during each iteration of model evaluation. The data preprocessing steps for the training and testing datasets are consistent, and the datasets used in the training and testing are different and non-overlapping.

#### Age-matched analysis in the HCC cohort

To further assess whether age differences influence HCC diagnosis, we constructed age-matched cohorts of individuals with and without HCC by resampling from the overlapping age range (47–71 years) shared by both groups. This overlapping range was defined as the intersection of the central 95% intervals of the age distributions in the HCC and non-HCC groups (Figure S6A). After age matching, we retained 60 non-HCC patients and 26 HCC patients, representing 74.1% of the original cohort. Figure S6B confirmed that there is no statistically significant age difference after age matching (*p* = 0.87, two-sided Mann-Whitney *U* test).

#### Cleavage proportion associated with 3′ end

We adapted the previously published methods for analyzing cleavage proportion in 5′ ends to 3' end analysis.^35^ In brief, we used the formula shown below for calculating cleavage proportion:

$$Cleavage\;proportion\;at\;a\;site\;i=\frac{No.\;of\;fragment\;ends\;at\;a\;site\;i}{Sequencing\;depth\;at\;site\;i}\times 100$$

where sequencing depth was defined as the number of sequence fragments covering a site *i*, and fragment ends refer to 3′ ends or 5' ends in this study. Cleavage profile was defined as the cleavage proportions across positions within a cleavage measurement window centered on a CpG. The window was defined as 5-nt upstream and downstream of a CpG. When we analyzed the cleavage profile of a number of cleavage measurement windows, the mean cleavage proportion of each relative position was used. As the CpG methylation at the Watson and Crick strands was often symmetrical, cleavage profiles of the Watson and Crick strands were merged in the 5′ to 3' direction for downstream analysis.

CpG sites were classified as hypermethylated if their methylation density consistently exceeded 70% across multiple tissue types, while those consistently below 30% were classified as hypomethylated. These tissue types include both normal tissues (i.e., buffy coat, liver, colon, kidney, prostate, urothelium, and placenta) and tumoral tissues (i.e., tumors from liver, colon, pancreas, kidney, prostate, and bladder). For differentially methylated CpG sites used for cancer detection, we included HCC-specific hypermethylated CpGs, and HCC-specific hypomethylated CpGs (i.e., a total of 4 types of CpG sites), as well as those hypomethylated CpG sites and hypermethylated CpG sites identified across various tissue types. The HCC-specific hypermethylated CpGs referred to CpGs with a methylation density of >70% in the HCC tumoral tissue and <30% in the buffy coat, and HCC-specific hypomethylated CpGs referred to CpGs with a methylation density of <30% in the HCC tumoral tissue and >70% in the buffy coat. Cleavage proportions in the cleavage profiles of these 4 types of CpG sites were input as features into the SVM model.

#### Definition of nuclease-specific end motifs

To study the engagement of nucleases on different ends for a single dsDNA molecule, the nuclease-specific end motifs were defined in terms of 2-mer end motifs for EM5 and EM3. Taking EM5 as an example, the 16 types of 2-mer end motifs were calculated and compared between wildtype (WT) mice and mice individually with the deletion of a nuclease such as *Dnase1l3*, *Dnase1* or *Dffb*. 2-mer end motifs exhibiting a significant reduction in frequency exclusively in a single nuclease knockout model were designated as nuclease-specific end motifs. As a result, we identified that 5′ end motif of GG and 3' end motif of CT were associated with DNASE1L3, with those 5′ end motif of CT and 3' end motif of CG for DNASE1, and those 5′ end motifs including AC, AT, AA, AG, and GA and 3' end motifs of GC, GT, AG, GG, and TC for DFFB. As a proof-of-concept study, we calculated the frequencies for EM5 and EM3 on the same side of the dsDNA molecules ($\frac{2\to 0}{2\leftarrow 0}$) in WT mice samples (observed motif frequency). We also calculated the frequencies for simulated $\frac{2\to 0}{2\leftarrow 0}$ motifs from the *in silico* randomly generated reads based on the size information of actual 4-end sequencing reads (expected motif frequency). To determine the relative abundance of the nuclease cutting, observed motif frequencies were normalized by the expected motif frequencies. In total, we analyzed 6 combinations of nuclease engagement by examining the proximal 5′ and 3' ends on the same side of a double-stranded cfDNA molecule. These combinations included DNASE1L3 (5′ end on one strand)–DNASE1L3 (3' end on the complementary strand), DNASE1–DNASE1, DFFB–DFFB, DNASE1–DNASE1L3, DFFB–DNASE1L3, and DFFB–DNASE1.

### Quantification and statistical analysis

Statistical analysis of motif rankings between different types of ends was conducted using the Pearson correlation test. For comparisons between two groups, statistical significance was assessed using a two-tailed Wilcoxon rank-sum test.

## Supplemental information

Document S1. Figures S1–S15 and Tables S1 and S3–S6

Table S2. Differential end motifs between advanced HCC (*n* = 10) and control (*n* = 38) groups using all plasma DNA fragments, related to Figure 3

Document S2. Article plus supplemental information
