# Supplementary material for: Holistic determination of ends of cfDNA molecules
Source: Cell Genom. 2026 Feb 6;6(3):101142. doi: 10.1016/j.xgen.2026.101142 (PMC12985390; doi:10.1016/j.xgen.2026.101142)
Supplement: Document S1. Figures S1–S15 and Tables S1 and S3–S6 [file mmc1.pdf]

Cell Genomics, Volume *6*

Supplemental information

Holistic determination of ends of cfDNA molecules

Peiyong Jiang, Mary-Jane L. Ma, Rong Qiao, Yuwei Shi, Jing Liu, Qing Zhou, Wenlei Peng, W.K. Jacky Lam, Jinyue Bai, L.Y. Lois Choy, W.H. Adrian Tsui, Yasine Malki, Guannan Kang, Stephanie C.Y. Yu, Dongyan Xiong, Grace L.H. Wong, Landon L. Chan, John Wong, Stephen L. Chan, Vincent W.S. Wong, K.C. Allen Chan, and Y.M. Dennis Lo

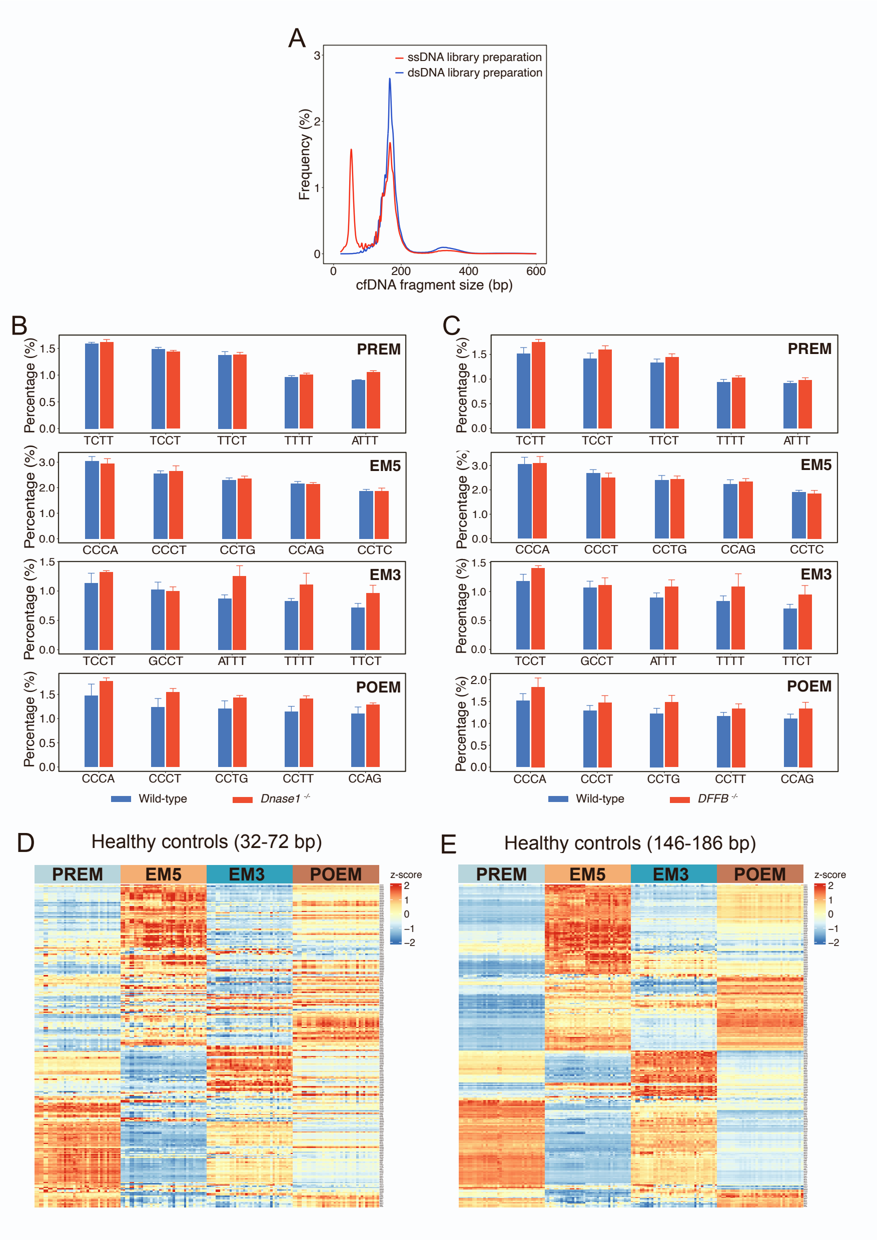

Figure S1. Size and end motif analyses for healthy human controls, related to Figure 2. (A) Size profiles of pooled sequencing results of plasma DNA from healthy controls using ssDNA library preparation or dsDNA library preparation. (B) Bar charts comparing motif frequencies between *DNASE1* knockout mice (n = 5) and matched wildtype mice (n = 5) for the top 5 motifs identified from healthy human controls (n = 38). In this bar plot, each bar represents the mean value, while the whiskers indicate one SD from the mean (the same below). (C) Bar charts comparing motif frequencies between *DFFB* knockout mice (n = 4) and matched wildtype mice (n = 6) for the top 5 motifs identified from healthy human controls. (D) Heatmap analysis of the 256 motif frequencies across PREM, EM5, EM3, and POEM using plasma DNA fragments ranging from 32 to 72 nt in healthy controls (n = 38). (E) Heatmap analysis of the 256 motif frequencies across PREM, EM5, EM3, and POEM using plasma DNA fragments ranging from 146 to 186 nt in healthy controls (n = 38). For better visualization, row-wise normalization (z-score) was applied to motif frequencies.

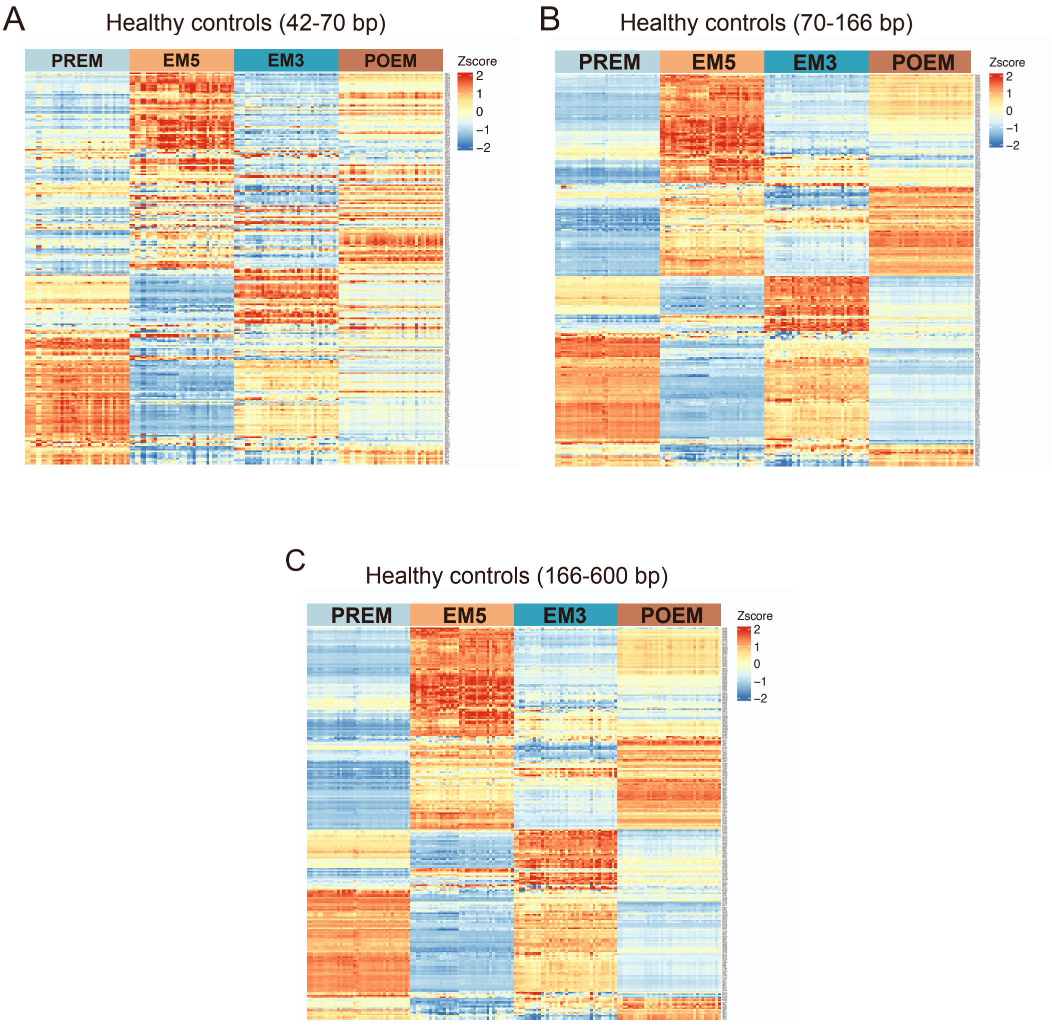

Figure S2. Size and end motif analyses for healthy human controls, related to Figure 3. Heatmap showed the 256 motif frequencies across PREM, EM5, EM3, and POEM using plasma DNA fragments with a size range of 42 to 70 nt (A), 70 – 166 nt (B), and 166 – 600 nt (C), respectively. For better visualization, row-wise normalization (z-score) was applied to motif frequencies. For each motif row, the mean (m) and standard deviation (s) of motif frequencies were computed across all individuals. Each individual motif frequency (f) was then standardized into a z-score (z) using the formula: $z = (f - m) / s$. Higher frequencies are represented in red, whereas lower frequencies are shown in blue.

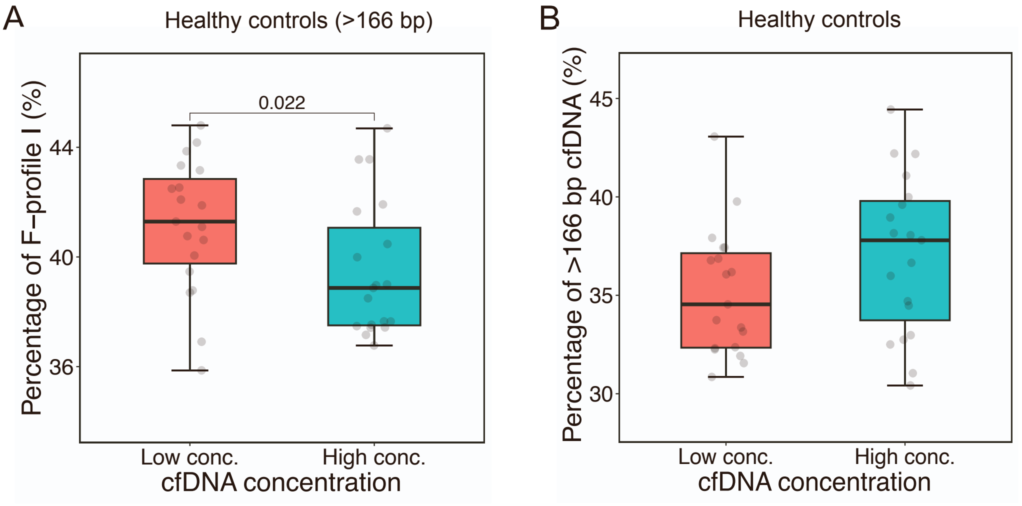

Figure S3. DNASE1L3 contribution and long cfDNA percentage in the low and high cfDNA concentrations using 2-end sequencing, related to Figure 3. (A) The F-profile I contribution (DNASE1L3 signature) based on the deconvolutional analysis of end motifs. In this boxplot, the central line shows the median value. The bottom and top edges of the box represent the 25th (Q1) and the 75th (Q2) percentiles. The whiskers extend to Q1 - 1.5x IQR and Q2 + 1.5 x IQR (the same below). (B) The proportion of long cfDNA fragments (> 166 bp).

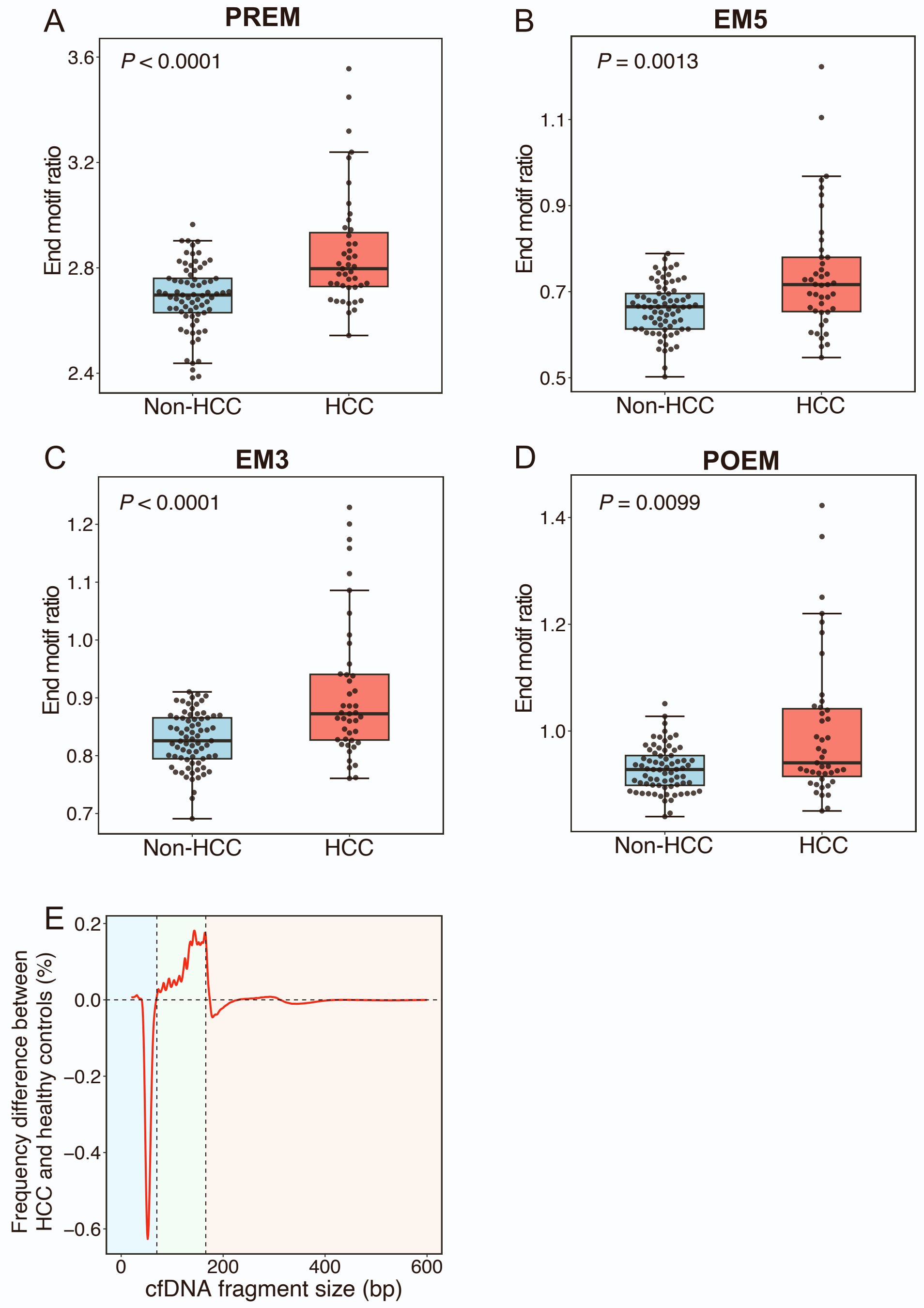

Figure S4. End motif ratio (EMR) analysis between HCC and non-HCC groups, related to Figure 3. Boxplots of end motif ratios were generated to compare the identified differential end motifs between HCC (n = 43) and non-HCC (n = 73) groups for PREM (A), EM5 (B), EM3 (C), and POEM (D). The differential end motifs were identified by comparing the motif frequencies between those patients with advanced HCC (n = 10) and healthy controls (n = 38). (E) The differences in size frequencies between pooled samples from HCC (n = 43) and control groups (n = 38).

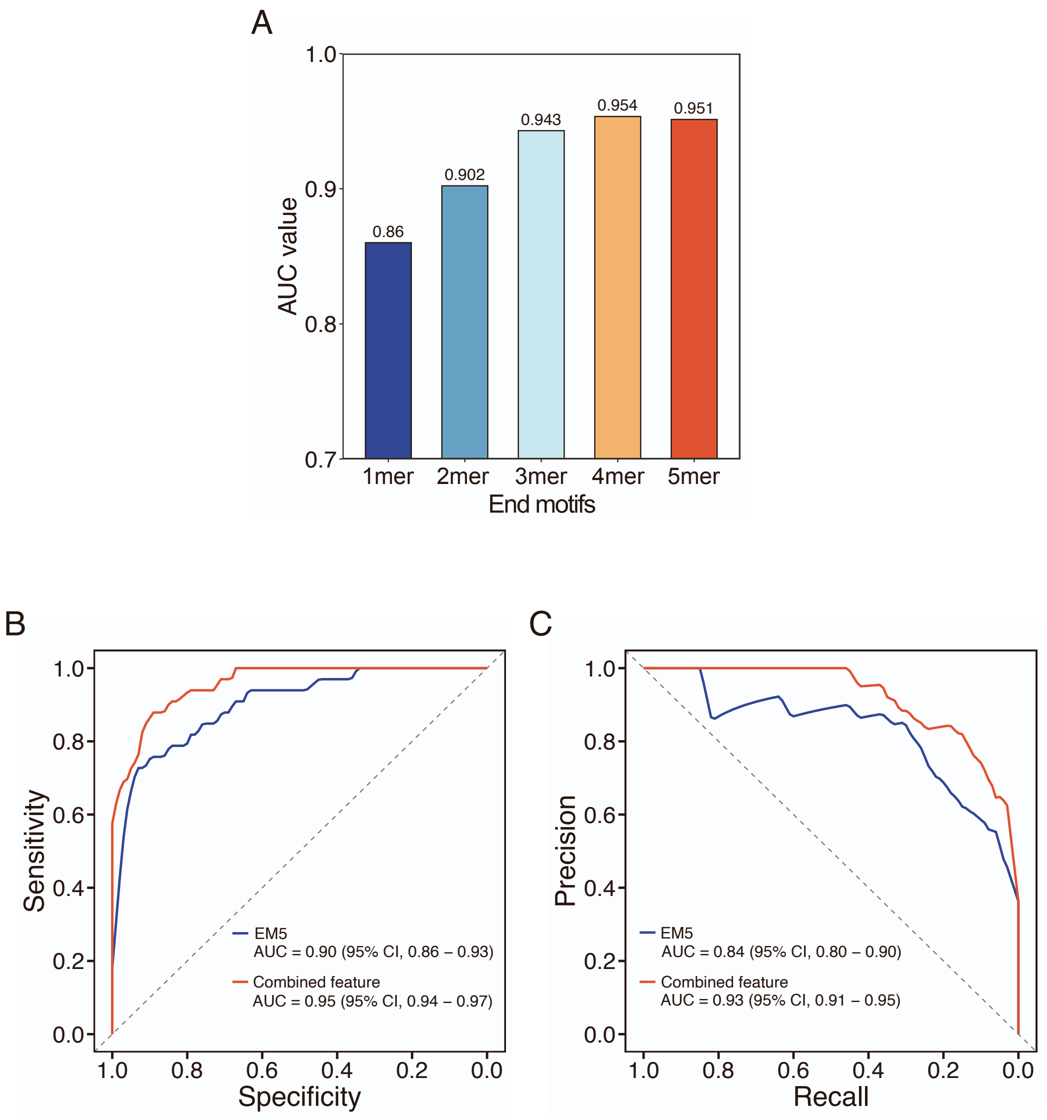

Figure S5. K-mer motif analysis and bootstrap analysis for model robustness with 1000 runs of resampling, related to Figure 4. (A) The AUC analysis for cancer detection using different k-mer motifs. (B) Receiver operating characteristic (ROC) curve analysis for EM5 and the combined feature set with 1000 runs of resampling using the 2-end sequencing dataset. (C) Precision-recall curve (PR) curve analysis for EM5 and the combined feature set with 1000 runs of resampling using 2-end sequencing dataset.

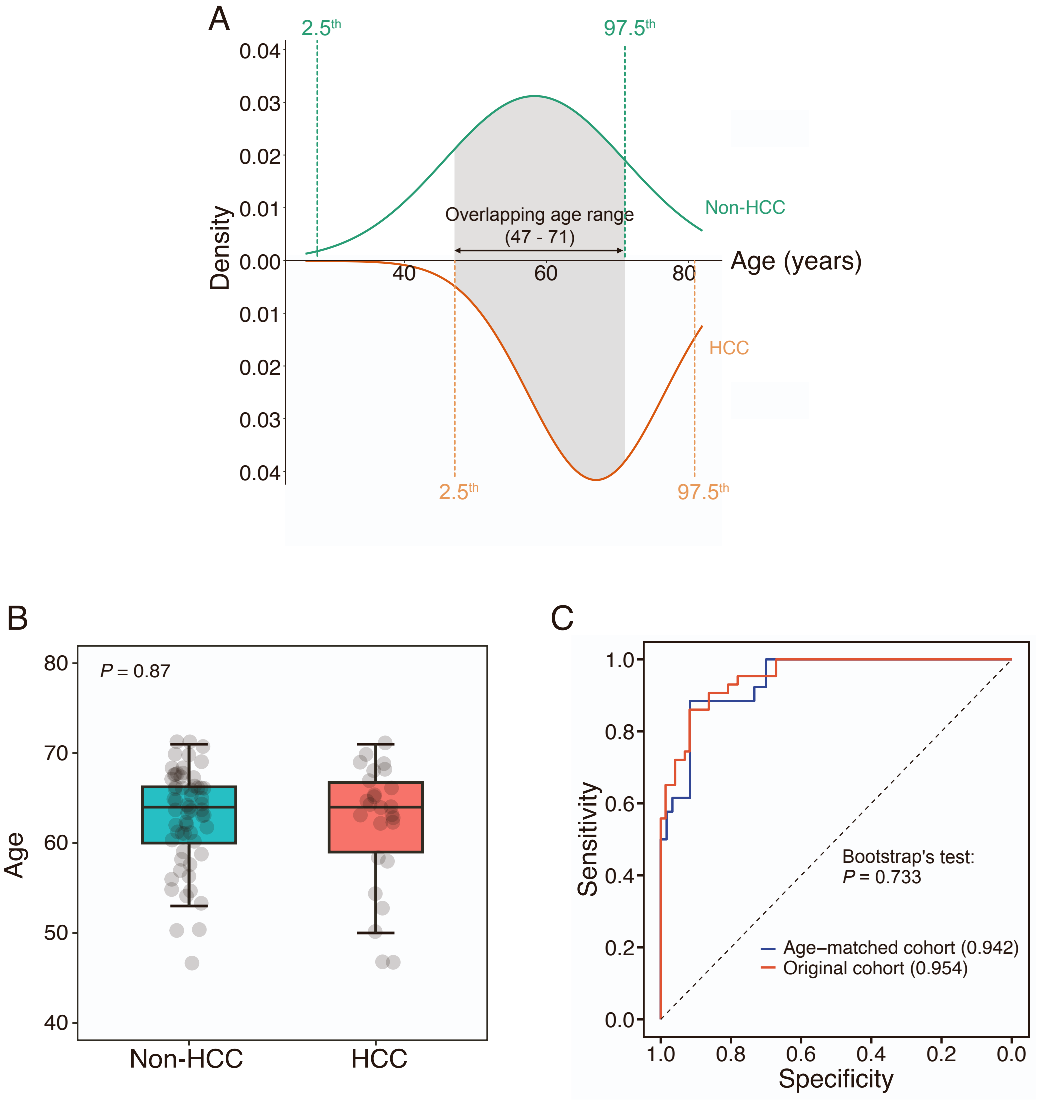

Figure S6. Age-matched analysis between HCC and non-HCC group, related to Figure 4. (A) Definition of overlapping age range between the non-HCC (n = 73) and HCC (n = 43) groups. This overlapping range was defined as the intersection of the central 95% intervals of the age distributions in the HCC and non-HCC groups. (B) The boxplot of ages between non-HCC (n = 60) and HCC (n = 26) groups after age-matching. (C) The ROC analysis for cancer detection for the age-matched cohort and the original cohort based on the combined analysis of size-stratified PREM, EM5, EM3 and POEM features.

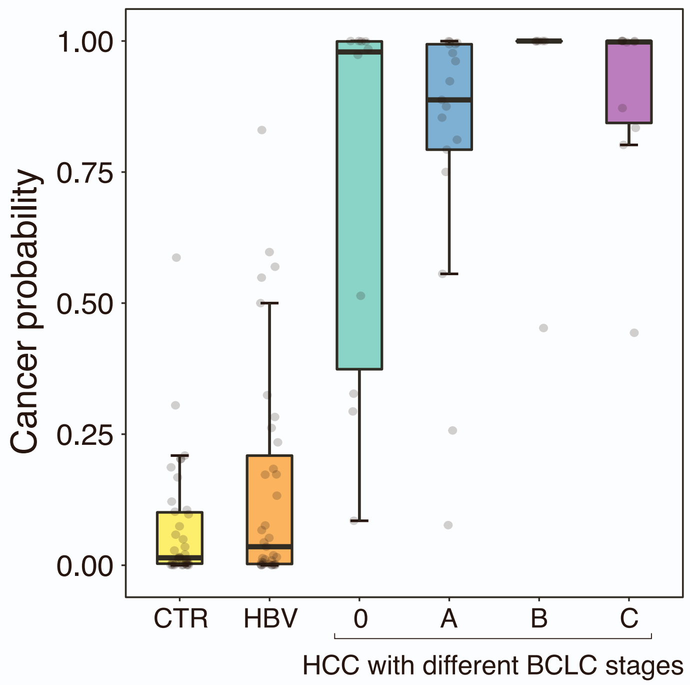

Figure S7. Boxplot of probabilities of having cancer using 3' FRAGMA features across healthy control, HBV, and HCC groups, related to Figure 5.

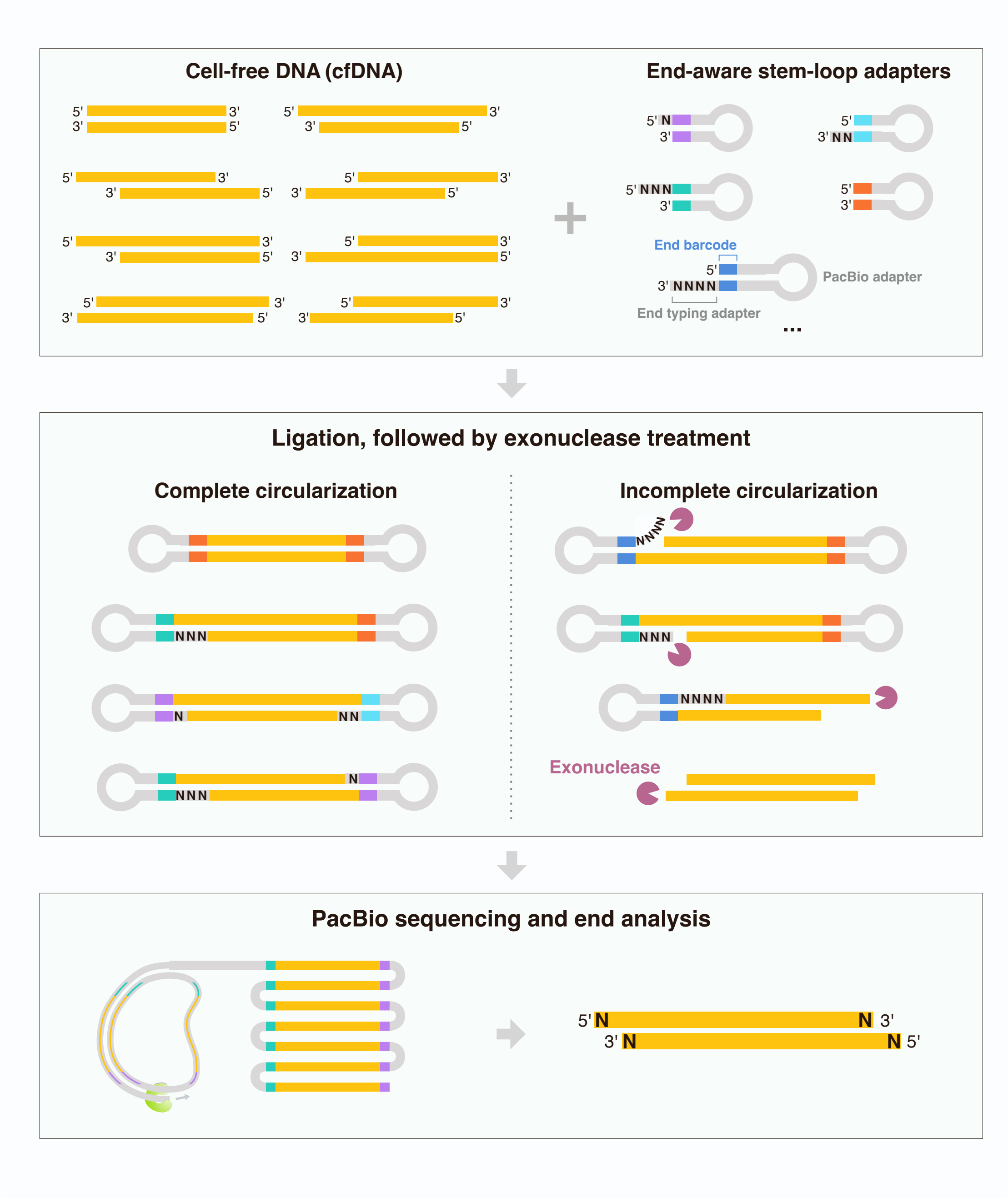

Figure S8. Schematic illustration of the cfDNA analysis using 4-end sequencing technology, related to Figure 6. Double-stranded cfDNA molecules, exhibiting various end modalities, are first ligated to the stem-loop adapters that have customized end structures capable of hybridizing to the matched native cfDNA ends. The end information, including length and base composition, is encoded in the sequence barcode within the stem region of the adapter. An additional step involving exonuclease treatment was implemented to eliminate incompletely ligated products, minimizing the risk of misreading barcode information caused by erroneous ligations. The circularized DNA molecules are then sequenced using single molecule real-time sequencing, SMRT-seq (Pacific Biosciences). In this method, both strands of the same molecule are sequenced multiple times, enabling the high-accurate decoding of all 4-end information for a single double-stranded molecule.

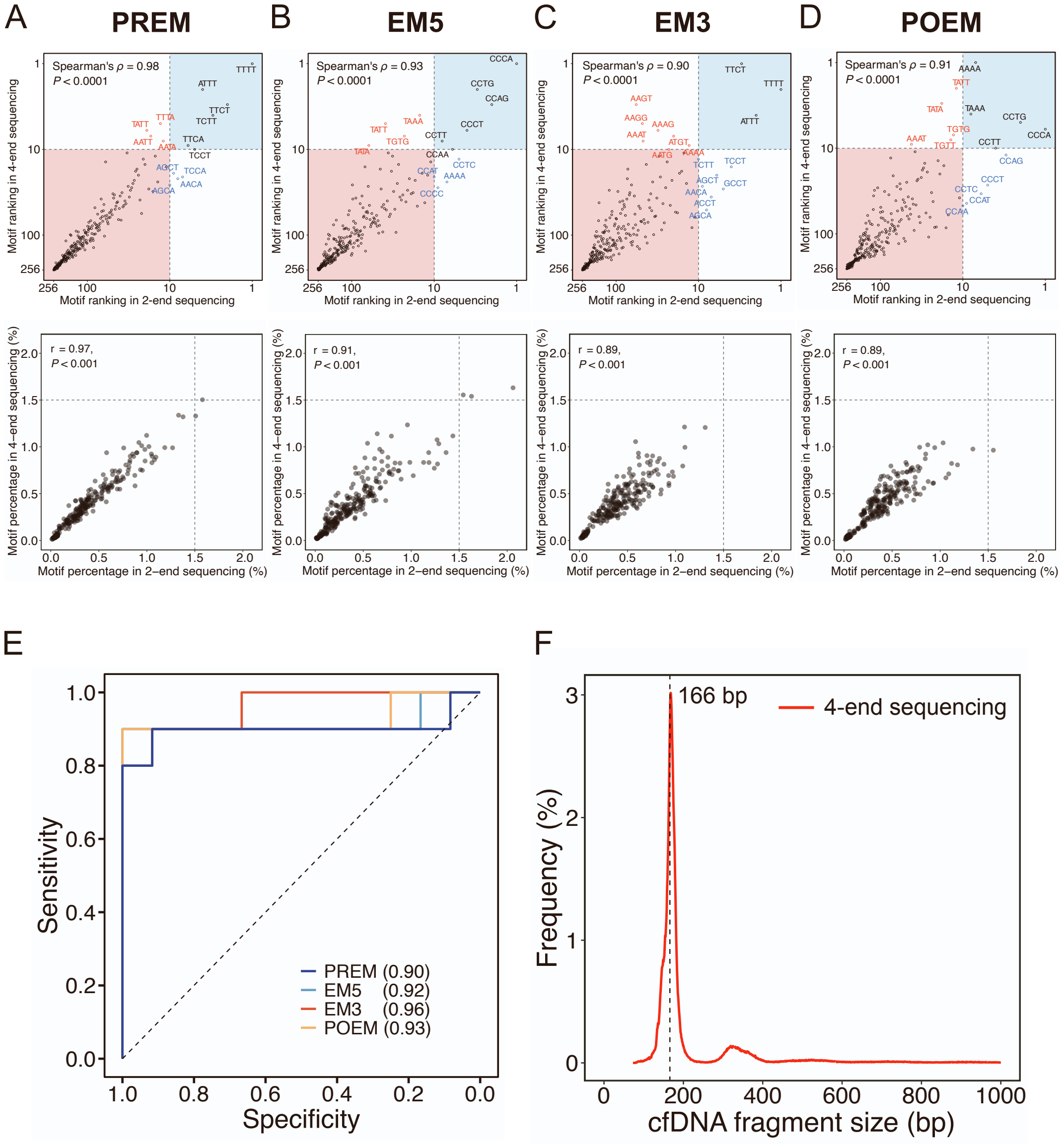

Figure S9. Comparisons of PREM, EM5, EM3, POEM and sizes between 2-end and 4-end sequencing results, related to Figures 2 and 6. Motif rankings and actual values of median frequencies of PREM (A), EM5 (B), EM3 (C), and POEM (D) between 2-end and 4-end sequencing results of plasma DNA of healthy controls. These 256 4-mer motifs were ranked in descending order based on their median motif frequencies among cfDNA samples. (E) ROC curves analysis of PREM, EM3, EM5, and POEM using SVM approach. (F) The size distribution of cfDNA sequenced using long-read sequencing (PacBio).

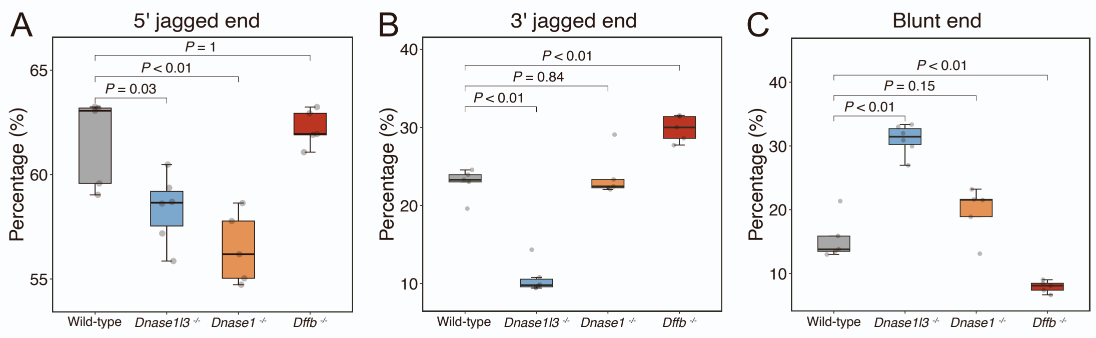

Figure S10. Comparisons of 5' jagged ends, 3' jagged end, and blunt end among wildtype mice and different types of knock-out mice, related to Figure 6. Percentage of the fragments carrying 5' jagged ends (A), 3' jagged ends (B), and blunt ends (C) among wildtype mice (n = 5), *DNASE1L3* knockout mice (n = 6), *DNASE1* knockout mice (n = 5), and *DFFB* knockout mice (n = 5).

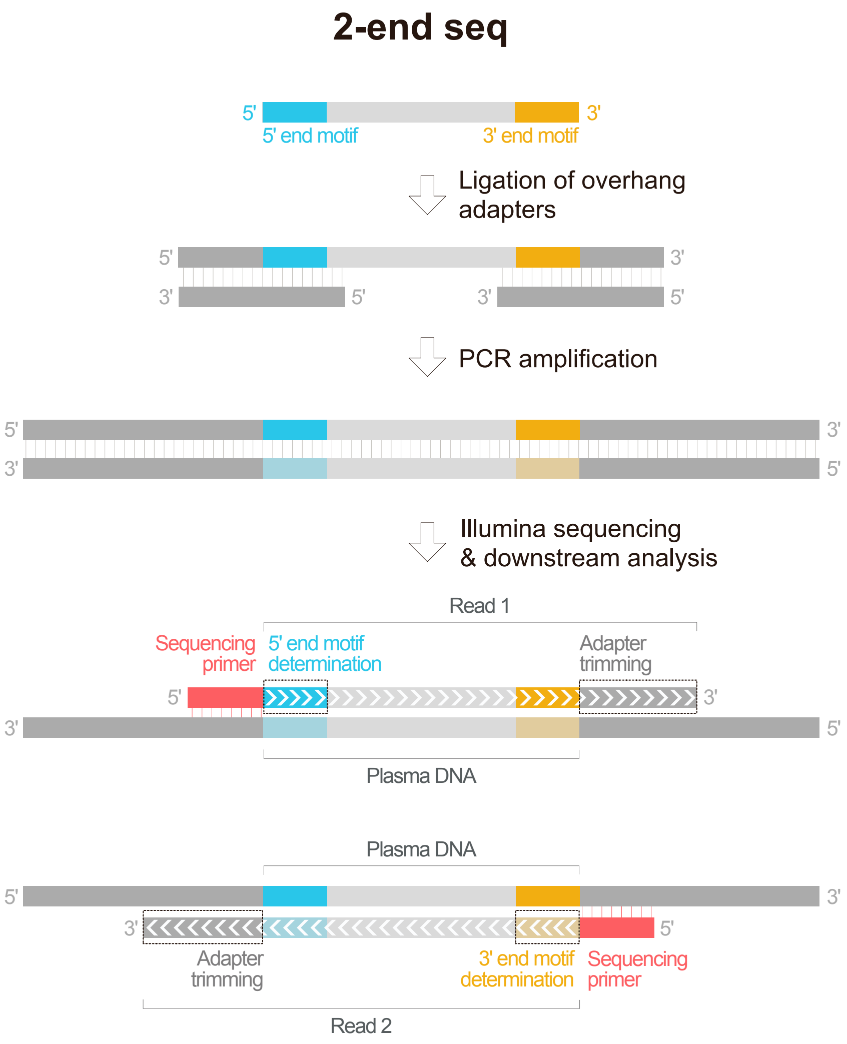

Figure S11. The adapter trimming methodology for 2-end sequencing, related to Figure 2. A single-stranded cfDNA molecule with native 5' and 3' end motifs (highlighted in blue and yellow, respectively) undergoes ligation with overhang adapters, followed by PCR amplification to generate double-stranded DNA. During Illumina paired-end sequencing, Read 1 is initiated by a sequencing primer (red) adjacent to the 3' end of the template strand, capturing the 5' end motif. Read 2 is similarly initiated by a sequencing primer (red) adjacent to the 3' end of the complementary strand, capturing the 3' end motif. In both reads, the number of sequencing cycles may extend beyond the length of the cfDNA insert, resulting in the inclusion of adapter sequences at the 3' ends. Therefore, adapter sequences are consistently found at the 3' ends of Read 1 and Read 2. Trimming the 3' ends of sequencing reads does not compromise the integrity of the native 5' termini, which are preserved at the beginning of each read.

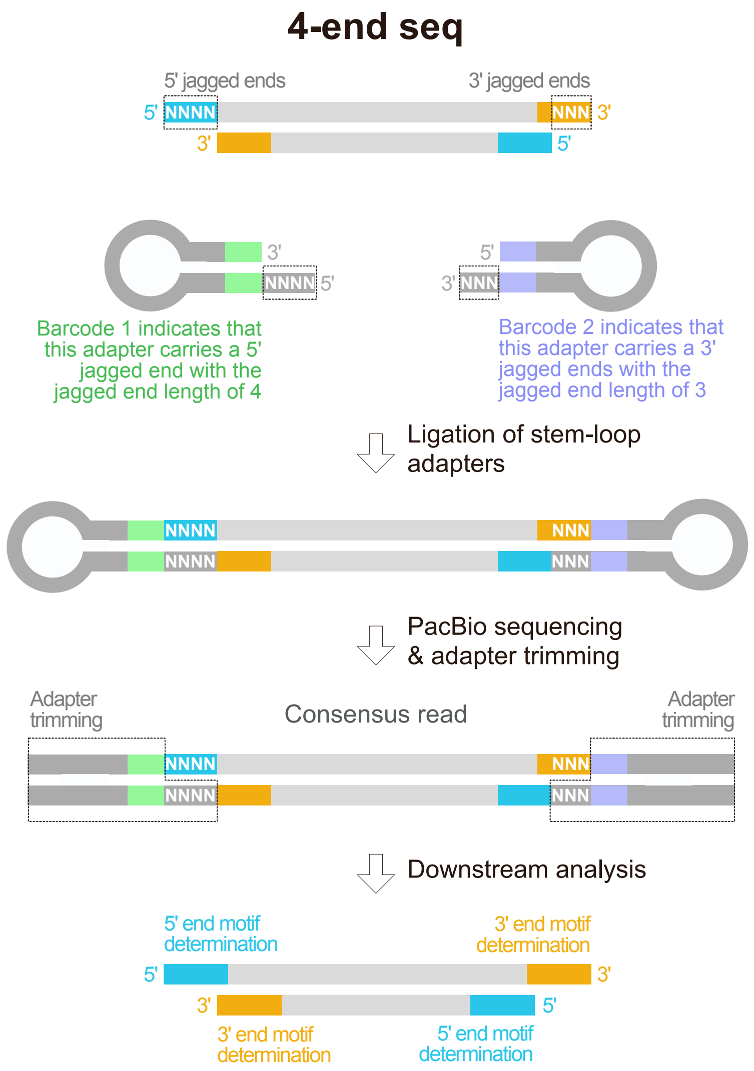

Figure S12. The adapter trimming methodology for 4-end sequencing, related to Figure 6. For illustration, we use a double-stranded cfDNA molecule that exhibits both 5' and 3' jagged ends as an example. More specifically, in this example, the double-stranded molecule carries a 5' overhang of four nucleotides ("NNNN") and a 3' overhang of three nucleotides ("NNN"). Stem-loop adapters containing Barcode 1 and Barcode 2 are designed to ligate to the 5' and 3' jagged ends, respectively, through perfect base pairing with the overhangs. Each barcode uniquely encodes structural information about the cfDNA termini, including the jagged end type (5' or 3') and jagged length. Following ligation, the cfDNA molecule is circularized and subjected to PacBio sequencing. Consensus sequences are generated for both the template and complementary strands. The barcode sequences, located near the loop regions, are first identified without nucleotide mismatches allowed. Based on this barcode information, we can accurately deduce the actual jagged end structures, such that the junctions between the cfDNA and adapter sequences on each strand can be precisely identified. Therefore, the adapter sequences are confidently located and removed, resulting in consensus reads that preserve all 4 native termini of a double-stranded cfDNA molecule.

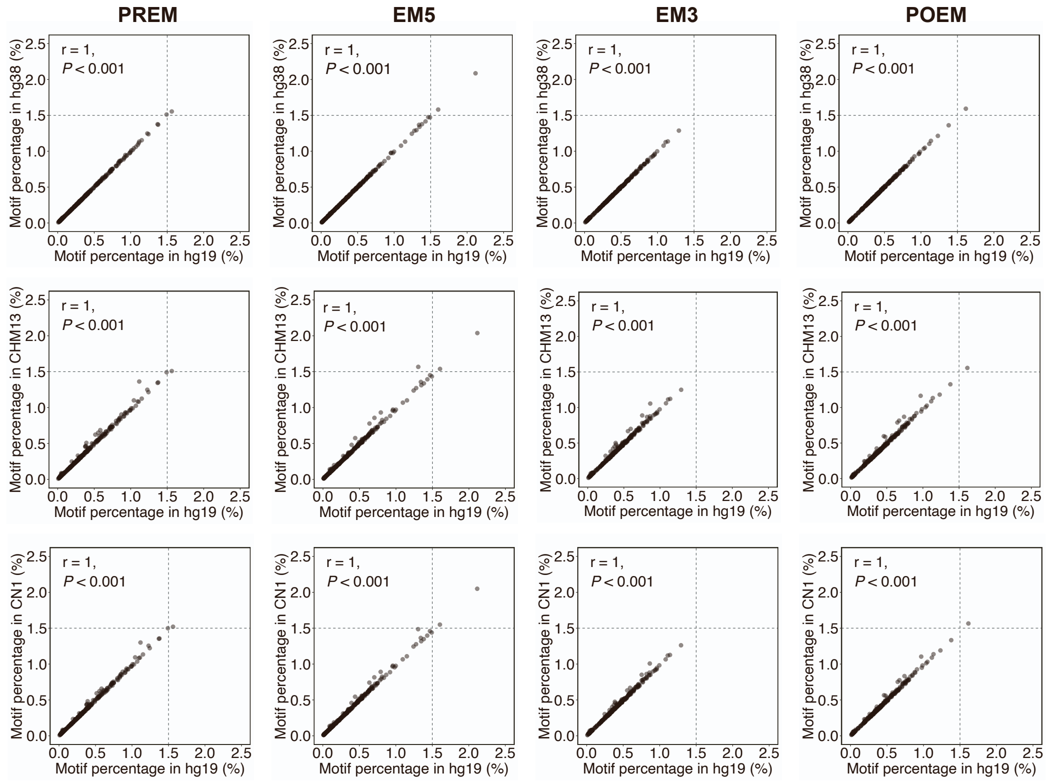

Figure S13. Motif consistency analysis across different versions of reference genomes, including hg38, CHM13, CN1, and hg19, related to Figure 2.

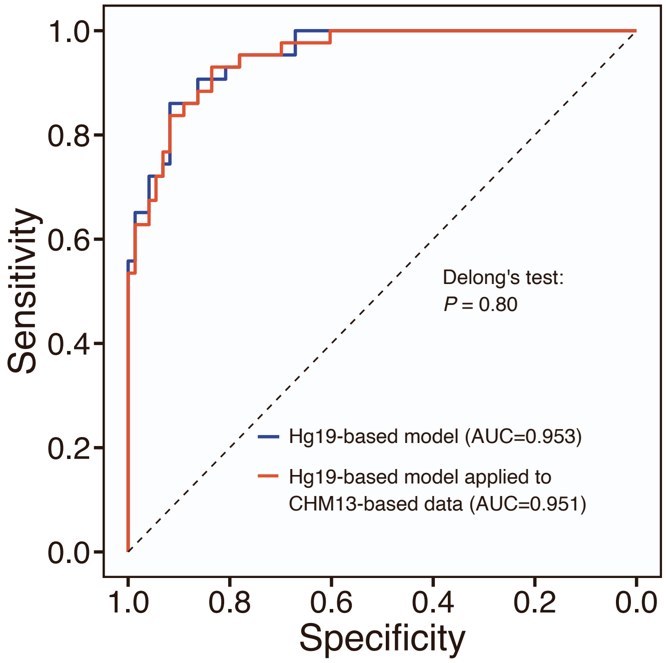

Figure S14. Receiver operating characteristic (ROC) analysis on the basis of different versions of human reference genomes, related to Figure 4.

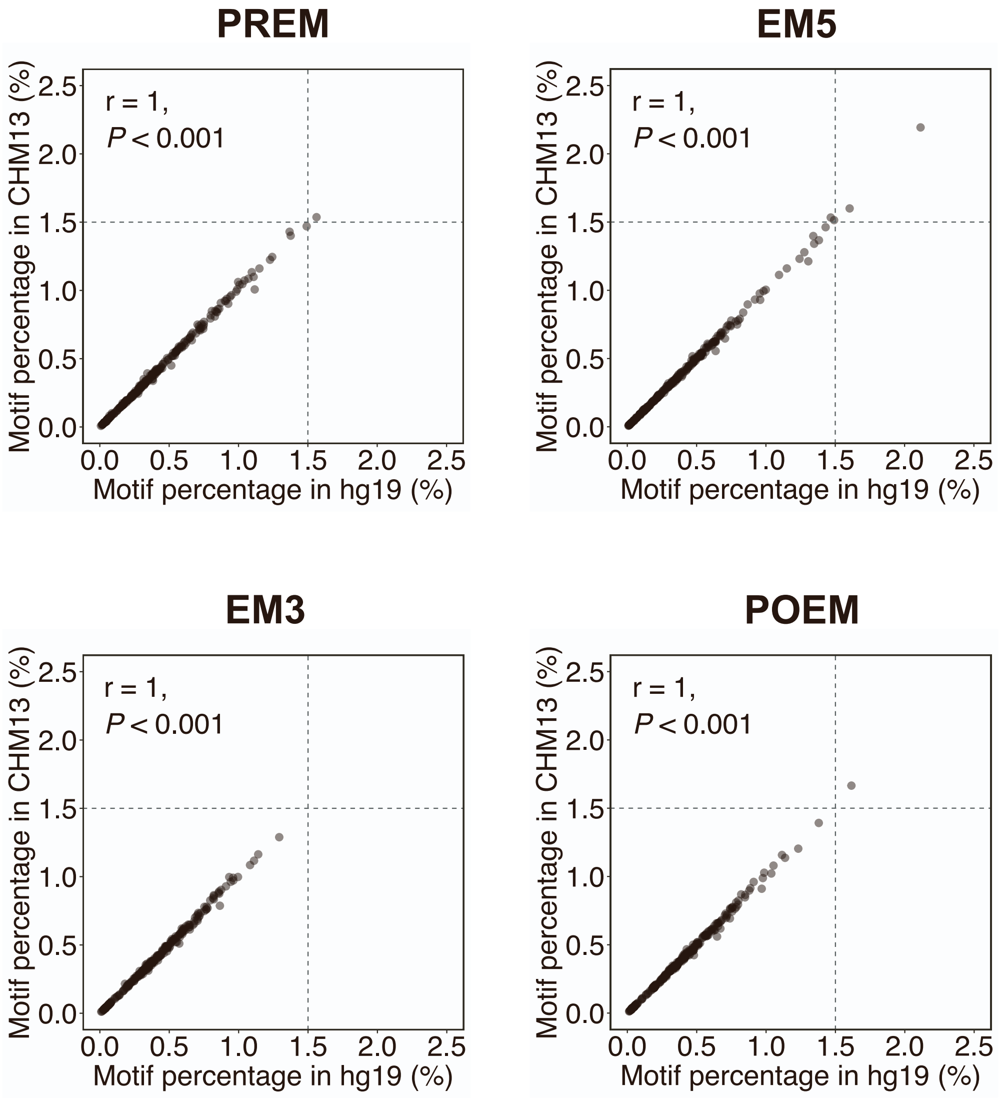

Figure S15. Scatter plots of motif frequencies between hg19 and CHM13 after *in silico* removal of sequenced fragments overlapping the centromeric, telomeric, and large segmental duplication regions, related to Figure 2.

Table S1. Top 20 motifs ranked by frequency for each type of end motif using all plasma DNA fragments in healthy human controls (n = 38), related to Figure 2.

Rank	PREM		EM5		EM3		POEM	
	Motif	Frequency (%)	Motif	Frequency (%)	Motif	Frequency (%)	Motif	Frequency (%)
1	TTTT	1.58	CCCA	2.06	TTTT	1.31	CCCA	1.55
2	TTCT	1.51	CCAG	1.63	ATTT	1.10	CCTG	1.34
3	TCTT	1.38	CCTG	1.54	TTCT	1.09	CCAG	1.18
4	ATTT	1.33	CCCT	1.43	TCCT	1.07	CCTT	1.12
5	TCCT	1.27	CCTC	1.36	GCCT	0.98	CCCT	1.09
6	TTCA	1.19	CCAA	1.29	AGCT	0.97	CCTC	1.06
7	TCCA	1.09	AAAA	1.27	ACCT	0.93	AAAA	1.03
8	AACA	1.08	CCTT	1.27	AGCA	0.93	TAAA	0.94
9	AGCT	1.06	CCCC	1.24	AACA	0.93	CCAT	0.93
10	AGCA	1.05	CCAT	1.19	TCTT	0.88	CCAA	0.93
11	GCCT	1.05	CAAA	1.18	TCCA	0.85	CAAA	0.88
12	AATA	1.02	CCAC	1.13	ACCA	0.85	TATT	0.87
13	TTTA	1.00	GCCA	1.02	AAAA	0.83	TGTG	0.85
14	ATCA	0.98	GCCT	0.99	TTCA	0.82	TGTT	0.85
15	ATCT	0.98	TAAA	0.96	AATT	0.81	CCAC	0.83
16	ACCA	0.95	GAAA	0.90	AATA	0.80	CCCC	0.79
17	AATT	0.95	GGAG	0.88	ATCT	0.78	TCTT	0.79
18	ACCT	0.93	GGCA	0.87	ACTT	0.76	TATA	0.79
19	TATT	0.92	GCTG	0.86	ATCA	0.76	GGAG	0.78
20	ATGT	0.90	CACA	0.83	ATGT	0.76	TCCT	0.77

Table S3. Top 20 motifs of PREM, EM5, EM3, and POEM in healthy controls between 2-end seq and 4-end sequencing results, related to Figures 2 and 6.

Rank	PREM				EM5				EM3				POEM			
	Motif	2-end seq (%)	Motif	4-end seq (%)	Motif	2-end seq (%)	Motif	4-end seq (%)	Motif	2-end seq (%)	Motif	4-end seq (%)	Motif	2-end seq (%)	Motif	4-end seq (%)
1	TTTT	1.58	TTTT	1.50	CCCA	2.06	CCCA	1.63	TTTT	1.31	TTCT	1.21	CCCA	1.55	AAAA	1.04
2	TTCT	1.51	ATTT	1.34	CCAG	1.63	CCTG	1.55	ATTT	1.10	TTTT	1.21	CCTG	1.34	TATT	1.02
3	TCTT	1.38	TTCT	1.33	CCTG	1.54	CCAG	1.54	TTCT	1.09	AAGT	1.06	CCAG	1.18	TATA	1.00
4	ATTT	1.33	TCTT	1.32	CCCT	1.43	TAAA	1.23	TCCT	1.07	ATTT	0.99	CCTT	1.12	TAAA	0.99
5	TCCT	1.27	TTTA	1.12	CCTC	1.36	TATT	1.14	GCCT	0.98	AAGG	0.93	CCCT	1.09	CCTG	0.98
6	TTCA	1.19	TATT	1.05	CCAA	1.29	CCCT	1.12	AGCT	0.97	AAAG	0.91	CCTC	1.06	CCCA	0.96
7	TCCA	1.09	AATT	1.01	AAAA	1.27	TGTG	1.11	ACCT	0.93	ATGT	0.90	AAAA	1.03	TGTG	0.92
8	AACA	1.08	AATA	1.00	CCTT	1.27	CCTT	1.08	AGCA	0.93	AAAT	0.89	TAAA	0.94	TGTT	0.87
9	AGCT	1.06	TTCA	0.99	CCCC	1.24	TATA	1.04	AACA	0.93	AAAA	0.88	CCAT	0.93	AAAT	0.83
10	AGCA	1.05	TCCT	0.99	CCAT	1.19	CCAA	1.03	TCTT	0.88	AATG	0.85	CCAA	0.93	CCTT	0.82
11	GCCT	1.05	TATA	0.99	CAAA	1.18	TGAG	0.97	TCCA	0.85	AATT	0.84	CAAA	0.88	TTTT	0.81
12	AATA	1.02	ATCT	0.97	CCAC	1.13	TGAA	0.95	ACCA	0.85	ATAT	0.83	TATT	0.87	CCAG	0.81
13	TTTA	1.00	AGTT	0.94	GCCA	1.02	CCTC	0.94	AAAA	0.83	TCTT	0.81	TGTG	0.85	ACTT	0.80
14	ATCA	0.98	AAGT	0.94	GCCT	0.99	CAAA	0.94	TTCA	0.82	AACT	0.81	TGTT	0.85	TCCT	0.79
15	ATCT	0.98	ATGT	0.94	TAAA	0.96	TGTT	0.89	AATT	0.81	TTGT	0.81	CCAC	0.83	TACA	0.78
16	ACCA	0.95	GCCT	0.92	GAAA	0.90	TAAT	0.88	AATA	0.80	TCCT	0.80	CCCC	0.79	TCTT	0.77
17	AATT	0.95	ATTA	0.92	GGAG	0.88	TACA	0.87	ATCT	0.78	GAGG	0.77	TCTT	0.79	TGAA	0.77
18	ACCT	0.93	AACT	0.90	GGCA	0.87	CAAG	0.84	ACTT	0.76	AATA	0.76	TATA	0.79	TAAT	0.75
19	TATT	0.92	AGCT	0.90	GCTG	0.86	GCCT	0.83	ATCA	0.76	ATCT	0.74	GGAG	0.78	TGAG	0.75
20	ATGT	0.90	TGTT	0.90	CACA	0.83	CCAC	0.83	ATGT	0.76	AGCT	0.73	TCCT	0.77	GGAG	0.74

Table S4. The sex and smoking information among non-cancer and cancer groups, related to Figure 4. F and M denote female and male, respectively; N and Y denote No and Yes, respectively.

Groups	Sex	Smoking
Individuals without cancer (n=73)	F: 14 (19.2%); M: 59 (80.8%)	N: 59 (90.8%); Y: 6 (9.2%)
Individuals with cancer (n=43)	F: 6 (14.0%); M: 37 (86.0%)	N: 31 (77.5%); Y: 9 (22.5%)

Table S5. Sequence information for customized stem-loop adapters used in 4-end sequencing, related to Figure 6.

Adapter	Sequence (5' -> 3')
Blunt	CGATGTATCTCTCTCTTTTCCTCCTCCTCCGTTGTTGTTGTTGAGAGAGATACATCG
5'_1 nt	NATCACGATCTCTCTCTTTTCCTCCTCCTCCGTTGTTGTTGTTGAGAGAGATCGTGAT
5'_2 nt	NNTTAGGCATCTCTCTCTTTTCCTCCTCCTCCGTTGTTGTTGTTGAGAGAGATGCCTAA
5'_3 nt	NNNTGACCAATCTCTCTCTTTTCCTCCTCCTCCGTTGTTGTTGTTGAGAGAGATTGGTCA
5'_4 nt	NNNNACAGTGATCTCTCTCTTTTCCTCCTCCTCCGTTGTTGTTGTTGAGAGAGATCACTGT
5'_5 nt	NNNNNGCCAATATCTCTCTCTTTTCCTCCTCCTCCGTTGTTGTTGTTGAGAGAGATATTGGC
5'_6 nt	NNNNNNCAGATCATCTCTCTCTTTTCCTCCTCCTCCGTTGTTGTTGTTGAGAGAGATGATCTG
5'_7 nt	NNNNNNNACTTGAATCTCTCTCTTTTCCTCCTCCTCCGTTGTTGTTGTTGAGAGAGATTCAAGT
5'_8 nt	NNNNNNNNGATCAGATCTCTCTCTTTTCCTCCTCCTCCGTTGTTGTTGTTGAGAGAGATCTGATC
5'_9 nt	NNNNNNNNNTAGCTTATCTCTCTCTTTTCCTCCTCCTCCGTTGTTGTTGTTGAGAGAGATAAGCTA
5'_10 nt	NNNNNNNNNNGGCTACATCTCTCTCTTTTCCTCCTCCTCCGTTGTTGTTGTTGAGAGAGATGTAGCC
5'_11 nt	NNNNNNNNNNNATGAGGATCTCTCTCTTTTCCTCCTCCTCCGTTGTTGTTGTTGAGAGAGATCCTCAT
5'_12 nt	NNNNNNNNNNNNGCAGAAATCTCTCTCTTTTCCTCCTCCTCCGTTGTTGTTGTTGAGAGAGATTTCTGC
5'_13 nt	NNNNNNNNNNNNNATCCACATCTCTCTCTTTTCCTCCTCCTCCGTTGTTGTTGTTGAGAGAGATGTGGAT
5'_14 nt	NNNNNNNNNNNNNNGCTTGTATCTCTCTCTTTTCCTCCTCCTCCGTTGTTGTTGTTGAGAGAGATACAAGC
5'_15 nt	NNNNNNNNNNNNNNNCAAGCTATCTCTCTCTTTTCCTCCTCCTCCGTTGTTGTTGTTGAGAGAGATAGCTTG
5'_16 nt	NNNNNNNNNNNNNNNNTGGATCATCTCTCTCTTTTCCTCCTCCTCCGTTGTTGTTGTTGAGAGAGATGATCCA
5'_17 nt	NNNNNNNNNNNNNNNNNAGTTCAATCTCTCTCTTTTCCTCCTCCTCCGTTGTTGTTGTTGAGAGAGATTGAACT
5'_18 nt	NNNNNNNNNNNNNNNNNNGACCTGATCTCTCTCTTTTCCTCCTCCTCCGTTGTTGTTGTTGAGAGAGATCAGGTC
5'_19 nt	NNNNNNNNNNNNNNNNNNNTCTCTAATCTCTCTCTTTTCCTCCTCCTCCGTTGTTGTTGTTGAGAGAGATTAGAGA
5'_20 nt	NNNNNNNNNNNNNNNNNNNNCTCTCGATCTCTCTCTTTTCCTCCTCCTCCGTTGTTGTTGTTGAGAGAGATCGAGAG
3'_1 nt	CTTGTAATCTCTCTCTTTTCCTCCTCCTCCGTTGTTGTTGTTGAGAGAGATTACAAGN
3'_2 nt	AGTCAAATCTCTCTCTTTTCCTCCTCCTCCGTTGTTGTTGTTGAGAGAGATTTGACTNN
3'_3 nt	AGTTCCATCTCTCTCTTTTCCTCCTCCTCCGTTGTTGTTGTTGAGAGAGATGGAACTNNN
3'_4 nt	ATGTCAATCTCTCTCTTTTCCTCCTCCTCCGTTGTTGTTGTTGAGAGAGATTGACATNNNN
3'_5 nt	CCGTCCATCTCTCTCTTTTCCTCCTCCTCCGTTGTTGTTGTTGAGAGAGATGGACGGNNNNN
3'_6 nt	GTCCGCATCTCTCTCTTTTCCTCCTCCTCCGTTGTTGTTGTTGAGAGAGATGCGGACNNNNNN
3'_7 nt	GTGAAAATCTCTCTCTTTTCCTCCTCCTCCGTTGTTGTTGTTGAGAGAGATTTTCACNNNNNNN
3'_8 nt	GTGGCCATCTCTCTCTTTTCCTCCTCCTCCGTTGTTGTTGTTGAGAGAGATGGCCACNNNNNNNN
3'_9 nt	GTTTCGATCTCTCTCTTTTCCTCCTCCTCCGTTGTTGTTGTTGAGAGAGATCGAAACNNNNNNNNN
3'_10 nt	CGTACGATCTCTCTCTTTTCCTCCTCCTCCGTTGTTGTTGTTGAGAGAGATCGTACGNNNNNNNNNN
3'_11 nt	CCAAGTATCTCTCTCTTTTCCTCCTCCTCCGTTGTTGTTGTTGAGAGAGATACTTGGNNNNNNNNNNN
3'_12 nt	TTGGACATCTCTCTCTTTTCCTCCTCCTCCGTTGTTGTTGTTGAGAGAGATGTCCAANNNNNNNNNNNN
3'_13 nt	CAGTAGATCTCTCTCTTTTCCTCCTCCTCCGTTGTTGTTGTTGAGAGAGATCTACTGNNNNNNNNNNNNN
3'_14 nt	GGCTTAATCTCTCTCTTTTCCTCCTCCTCCGTTGTTGTTGTTGAGAGAGATTAAGCCNNNNNNNNNNNNNN
3'_15 nt	TGACGAATCTCTCTCTTTTCCTCCTCCTCCGTTGTTGTTGTTGAGAGAGATTCGTCANNNNNNNNNNNNNNN
3'_16 nt	AATCCGATCTCTCTCTTTTCCTCCTCCTCCGTTGTTGTTGTTGAGAGAGATCGGATTNNNNNNNNNNNNNNNN
3'_17 nt	TAATACATCTCTCTCTTTTCCTCCTCCTCCGTTGTTGTTGTTGAGAGAGATGTATTANNNNNNNNNNNNNNNNN
3'_18 nt	CGGCGTATCTCTCTCTTTTCCTCCTCCTCCGTTGTTGTTGTTGAGAGAGATACGCCGNNNNNNNNNNNNNNNNNN
3'_19 nt	ATGTAAATCTCTCTCTTTTCCTCCTCCTCCGTTGTTGTTGTTGAGAGAGATTTACATNNNNNNNNNNNNNNNNNNN
3'_20 nt	GCACGGATCTCTCTCTTTTCCTCCTCCTCCGTTGTTGTTGTTGAGAGAGATCCGTGCNNNNNNNNNNNNNNNNNNNN

Table S6. Top 5 most deviated 4-end motifs in each motif category between hg19 and CHM13, related to Figure 2.

Categories	Top 5 motifs in each category	Motif frequencies (%, hg19)	Motif frequencies (%, CHM13)	Deviation (%)
PREM	TCCA	1.12	1.36	0.25
	ATTC	0.39	0.51	0.12
	TTCC	0.52	0.62	0.11
	AATG	0.58	0.69	0.1
	CATT	0.55	0.65	0.1
EM5	CCAT	1.31	1.57	0.26
	GGAA	0.71	0.86	0.15
	TCCA	0.64	0.78	0.14
	CATT	0.79	0.93	0.14
	GAAT	0.44	0.57	0.13
EM3	TCCA	0.86	1.06	0.19
	AATG	0.75	0.89	0.13
	ATTC	0.39	0.5	0.11
	ATGG	0.56	0.67	0.11
	CATT	0.59	0.7	0.1
POEM	CCAT	0.97	1.16	0.2
	GGAA	0.67	0.82	0.14
	TCCA	0.65	0.79	0.14
	GAAT	0.46	0.6	0.14
	CATT	0.74	0.87	0.13
